# Irreversible entropy transport enhanced by fermionic superfluidity

**DOI:** 10.1038/s41567-024-02483-3

**Published:** 2024-04-22

**Authors:** Philipp Fabritius, Jeffrey Mohan, Mohsen Talebi, Simon Wili, Wilhelm Zwerger, Meng-Zi Huang, Tilman Esslinger

**Affiliations:** 1 Institute for Quantum Electronics & Quantum Center, ETH Zurich, Zurich, Switzerland; 2 Physik Department, Technische Universität München, Garching, Germany

**Keywords:** Bose-Einstein condensates, Quantum fluids and solids, Phase transitions and critical phenomena

## Abstract

The nature of particle and entropy flow between two superfluids is often understood in terms of reversible flow carried by an entropy-free, macroscopic wavefunction. While this wavefunction is responsible for many intriguing properties of superfluids and superconductors, its interplay with excitations in non-equilibrium situations is less understood. Here we observe large concurrent flows of both particles and entropy through a ballistic channel connecting two strongly interacting fermionic superfluids. Both currents respond nonlinearly to chemical potential and temperature biases. We find that the entropy transported per particle is much larger than the prediction of superfluid hydrodynamics in the linear regime and largely independent of changes in the channel’s geometry. By contrast, the timescales of advective and diffusive entropy transport vary significantly with the channel geometry. In our setting, superfluidity counterintuitively increases the speed of entropy transport. Moreover, we develop a phenomenological model describing the nonlinear dynamics within the framework of generalized gradient dynamics. Our approach for measuring entropy currents may help elucidate mechanisms of heat transfer in superfluids and superconducting devices.

## Abstract (web summary)

Connecting two superfluid reservoirs leads to both particle and entropy flow between the systems. Now, a direct measurement of the entropy current and production in ultracold quantum gases reveals how superfluidity enhances entropy transport.

## Main

Two connected reservoirs exchanging particles and energy is a paradigmatic system that is key to understanding transport phenomena in diverse platforms of both fundamental and technological interest ranging from heat engines to superconducting qubits^1^ and even heavy-ions collisions^2^. Entropy and heat, both irreversibly produced and transported by the currents flowing between the reservoirs, are key quantities in superfluid and superconducting systems^3,4^. They help to reveal microscopic information in strongly interacting systems^5,6^ and more generally characterize far-from-equilibrium systems^7^. Yet in traditional condensed matter systems such as superconductors and superfluid helium, the entropy is not directly accessible and requires indirect methods to deduce it^8–10^.

In this work, we leverage the advantage of quantum gases of ultracold atoms as naturally closed systems well-isolated from their environments to study entropy transport and production in fermionic superfluid systems. Using the known equation of state^11^, we measure the particle number and total entropy in each of the two connected reservoirs as a function of evolution time, therefore directly obtaining the entropy current and production. In general, the nature of these currents depends on the coupling strength between the superfluids. On the one hand, two weakly coupled superfluids exhibiting the Josephson effect^12,13^ exchange an entropy-free supercurrent described by Landau’s hydrodynamic two-fluid model^14–18^. In quantum gases^19^ as well as superconductors^20^, this is accomplished with low-transparency tunnel junctions weakly biased in chemical potential or phase, while narrow channels are used to block viscous currents in superfluid helium^21,22^. On the other hand, superfluids strongly coupled by high-transparency channels^23^ can exhibit less intuitive behaviour since the supercurrent no longer dominates the normal current, making the system fundamentally non-equilibrium^24,25^. In particular, the signature of superfluidity in such systems is often large particle currents on the order of the superfluid gap which respond nonlinearly to chemical potential biases smaller than the gap. This is observed in ballistic junctions between superconductors^20^, superfluid He^26^ and quantum gases^27–29^. However, entropy transport in this setting has so far only been experimentally studied indirectly and at higher temperatures in the linear response regime where the superfluidity of the system is ambiguous^30,31^, leaving open the question of entropy transport between strongly coupled superfluids.

Here, we connect two superfluid unitary Fermi gases with a ballistic channel and measure the coupled transport of particles and entropy between them. We observe large subgap currents of both particles and entropy, indicating that the current is not a pure supercurrent and cannot be understood within a hydrodynamic two-fluid model. In particular, superfluidity counterintuitively enhances entropy transport in this system by enhancing particle current while maintaining a large entropy transported per particle. We also observe in our parameter regime that, while the system can always thermalize via the irreversible flow of this superfluid-enhanced normal current, thermalization via pure entropy diffusion is inhibited in one-dimensional (1D) channels, giving rise to a non-equilibrium steady state previously observed in the normal phase^30^. The observed nonlinear dynamics of particles and entropy are captured by a phenomenological model we develop whose only external constraints are the conservation of particles and energy and the second law of thermodynamics.

**Fig. 1:**
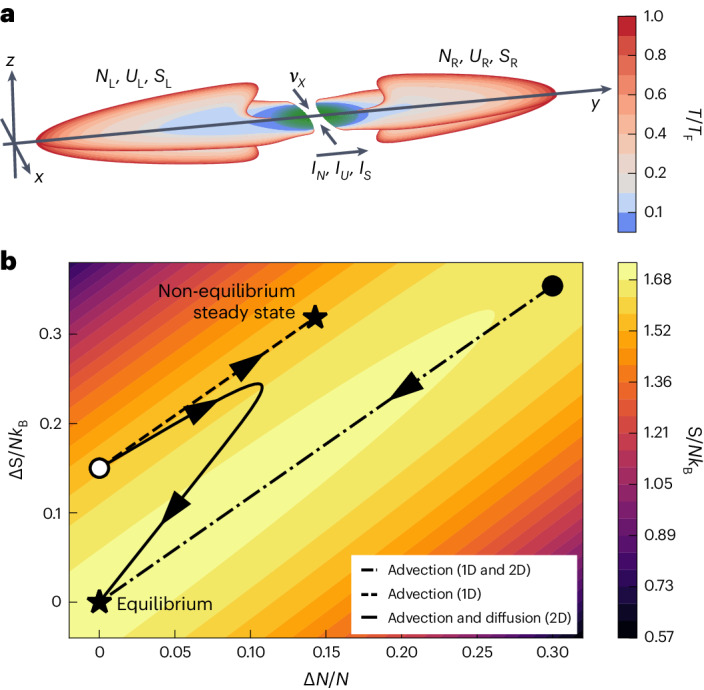
Irreversible particle, energy and entropy transport through a non-equilibrium channel connecting two superfluids. **a**, Slices along the *x* = 0 and *z* = 0 planes of the calculated local degeneracy *T*/*T*_F_ during transport through the 1D channel, showing that the reservoirs (left L and right R) are in local equilibrium in the normal phase (red, *T*/*T*_F_ > 0.167) over most of their volume and superfluid (blue, *T*/*T*_F_ ≤ 0.167) at the contacts to the channel. The channel (green) is far from local equilibrium. Differences in the atom number *N*, energy *U* and entropy *S* between the reservoirs induce currents between them. **b**, Depending on the initial conditions (filled or open circles) and microscopically allowed processes, the reservoirs can exchange entropy advectively and diffusively, tracing out various paths through state space with the constraints that atom number and energy are conserved d*N*/d*t* = d*U*/d*t* = 0 and the entropy production is positive definite d*S*/d*t* ≥ 0. This evolution halts (stars) by reaching either a non-equilibrium steady state (Δ*N*, Δ*S* ≠ 0) or equilibrium (Δ*N* = Δ*S* = 0) where the total entropy *S* = *S*_L_ + *S*_R_ has a global maximum.

We begin the experiment by preparing a balanced mixture of the first- and third-lowest hyperfine ground states of ^6^Li at unitarity in an augmented harmonic trap shown in Fig. 1a (Methods and Supplementary Information section 3). To induce transport of atoms, energy and entropy between the left (L) and right (R) reservoirs, we prepare an initial state within the state space in Fig. 1b characterized by the conserved total atom number and energy, *N* = *N*_L_ + *N*_R_ and *U* = *U*_L_ + *U*_R_, and the dynamical imbalances in the atom number Δ*N* = *N*_L_ − *N*_R_ and entropy Δ*S* = *S*_L_ − *S*_R_. The imbalances in the extensive quantities induce biases in the chemical potential Δ*μ* = *μ*_L_ − *μ*_R_ and temperature Δ*T* = *T*_L_ − *T*_R_ according to the reservoirs’ equations of state (EoS; Methods) which in turn drive currents of the extensive properties *I*_*N*_(Δ*N*, Δ*S*) = − (1/2)dΔ*N*/d*t* and *I*_*S*_(Δ*N*, Δ*S*) = − (1/2)dΔ*S*/d*t*. Note that *I*_*S*_ is an apparent current, not a conserved current like *I*_*N*_ and *I*_*U*_, though we can place bounds on the conserved entropy current from the apparent current and entropy production rate ${I}_{S}^{{{\;{\rm{cons}}}}}\in [{I}_{S}-(1/2){{{\rm{d}}}}S/{{{\rm{d}}}}t,{I}_{S}+(1/2){{{\rm{d}}}}S/{{{\rm{d}}}}t]$ (ref. ^29^). These equations of motion, together with the initial state Δ*N*(0), Δ*S*(0), determine the path the system traces through state space Δ*N*(*t*), Δ*S*(*t*) as well as the speed with which it traces this path. The paths that we explore experimentally are shown as black lines overlayed on top of the entropy landscape *S* = *S*_L_(*N*_L_, *U*_L_) + *S*_R_(*N*_R_, *U*_R_) = *S*(*N*, *U*, Δ*N*, Δ*S*) computed from the EoS. The paths all exhibit a strictly positive entropy production rate d*S*/d*t* > 0, indicating that the transport is irreversible, until they reach either a non-equilibrium steady state (Δ*N*, Δ*S* ≠ 0) or equilibrium (Δ*N* = Δ*S* = 0) where *S* is maximized for the fixed *N* and *U*. The von Neumann entropy of a closed system does not increase in time under Hamiltonian evolution, though the measured thermodynamic entropy *S* can increase due to buildup of entanglement entropy shared between the two reservoirs^32–35^. We have verified that the system is nearly closed given the measured particle loss rate ∣d*N*/d*t*∣/*N* < 0.01 s^−1^ and heating in equilibrium d(*S*/*Nk*_B_)/d*t* < 0.02 s^−1^, limited by photon scattering of optical potentials, such that the entropy production observed during transport is due to the fundamental irreversibility of the transport.

We measure the evolution of the system by repeatedly preparing the system in the same initial state, allowing transport for a time *t*, then taking absorption images of both spin states and extracting *N*_*i*_, *S*_*i*_ for both reservoirs *i* = L, R using standard thermometry techniques (Methods and Supplementary Information section 5B). Between the end of transport and imaging, we adiabatically ramp down the laser beams that define the channel to image the reservoirs in well-calibrated, half-harmonic traps. The beams do work on the reservoirs during this process and change *U*_*i*_ but *N*_*i*_ and *S*_*i*_ remain constant. The cloud typically contains *N* = 270(30) × 10^3^ atoms and *S*/*N* = 1.59(7)*k*_B_ before transport, below the critical value of ~1.90*k*_B_ for superfluidity in the transport trap (numbers in brackets represent statistical uncertainties). Figure 1a shows the local degeneracy *T*/*T*_F_ in the *x* = 0 and *z* = 0 planes during transport calculated within the local density approximation^36^ using the three-dimensional (3D) equation of state^11^ to determine the local Fermi temperature *T*_F_(*x*, *y*, *z*) (Methods). The imbalance is illustrated using the representative values of *ν*_*x*_ = 12.4 kHz, Δ*μ* = 75 nK × *k*_B_ and Δ*T* = 0. Assuming local equilibrium, the most degenerate regions in the channel would be deeply superfluid due to the strong, attractive gate potential and reach *T*/*T*_F_ ≈ 0.027, *s* ≈ 7.2 × 10^−4^*k*_B_ and *Δ*/*k*_B_*T* ≈ 17 where *s* is the local entropy per particle and *Δ* is the superfluid gap. When *ν*_*x*_ ≲ 7 kHz (Methods and Supplementary Information Section 3) and the channel is two-dimensional (2D), normal regions appear at the edges of the channel that can also contribute to transport.

**Fig. 2:**
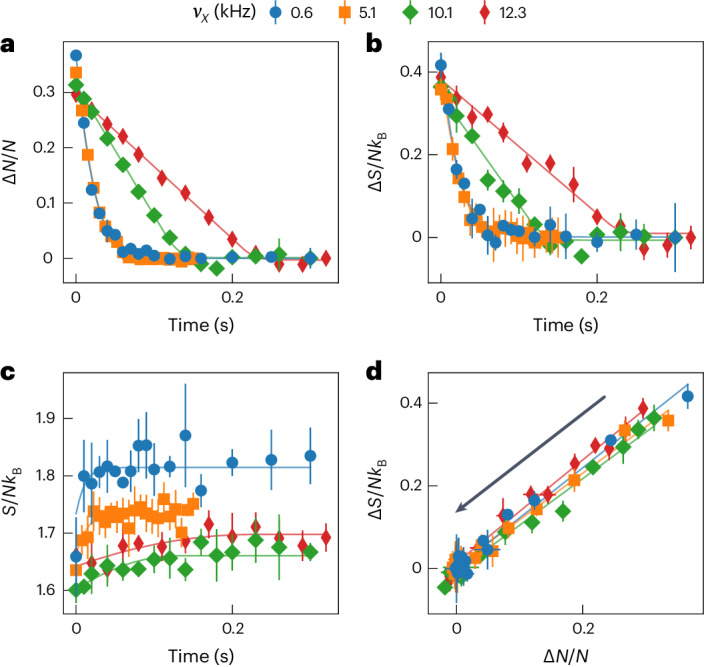
Observing advective entropy current from the non-exponential evolution of imbalances in particle number and entropy. **a**,**b**, Particles (**a**) and entropy (**b**) are transported between the reservoirs by currents that respond nonlinearly to biases in chemical potential and temperature, evidenced by the non-exponential decay. Both currents increase as the channel opens from 1D to 2D (*ν*_*x*_ decreasing). **c**, The net entropy increases very slightly while Δ*S* changes significantly, indicating that entropy is indeed transported by the particle flow. **d**, Showing *I*_*S*_ = s^*^I_*N*_ where the entropy advectively transported per particle s^*^ is nearly independent of the channel geometry *ν*_*x*_ despite *I*_*N*_ varying significantly. The observation that s^*^ > 0 means the nonlinear particle current is not a superfluid current. Each data point is an average of three to five repetitions and error bars represent the standard deviation. Solid lines are fits of the phenomenological model. Source data

In a first experiment, we prepare an initial state Δ*N*(0), Δ*S*(0) ≠ 0 (filled circle in Fig. 1b) such that equilibrium is reached within 1 s. For the strongest confinement, Δ*N*(*t*), shown in Fig. 2a, clearly deviates from exponential relaxation and the corresponding *I*_*N*_ is much larger than the value ~Δ*μ*/*h* of a quantum point contact in the normal state, where *h* is Planck’s constant, indicating that the subgap current-bias characteristics are nonlinear (non-Ohmic) and the reservoirs are superfluid^28,29,37^. When reducing *ν*_*x*_ to cross over from a 1D to 2D channel, Δ*N*(*t*) relaxes faster (*I*_*N*_ increases) and, although it is less pronounced, the nonlinearity persists. The dynamics for the two smallest values of *ν*_*x*_ are nearly identical, suggesting that there are additional resistances in series with the 1D region such as the viscosity of the bulk reservoirs or the interfaces between the 3D and 2D regions^31^ or between the normal and superfluid regions^38^.

Concurrently with Δ*N*(*t*), we observe non-exponential relaxation of Δ*S*(*t*) (Fig. 2b) which bears a remarkable resemblance to Δ*N*(*t*). We find that by plotting Δ*S*(*t*) against Δ*N*(*t*) in Fig. 2d, all paths collapse onto a single line. This demonstrates that the entropy current is directly proportional to the particle current *I*_*S*_ = s^*^I_*N*_ where the average entropy advectively transported per particle s^*^ is nearly independent of *ν*_*x*_ even though *I*_*N*_ itself varies significantly. Moreover, d*S*/d*t* (Fig. 2c) is barely resolvable and is significantly smaller than *I*_*S*_, meaning there is indeed a large conserved entropy current flowing between the reservoirs. The dependence of *S*/*Nk*_B_ on the confinement *ν*_*x*_ in Fig. 2c has a technical origin and does not affect the system during transport (Methods). The entropy transported per particle s^*^ = 1.18(3)*k*_B_ is near its value in the normal phase^30^ and is orders of magnitude larger than the local entropy per particle in the channel assuming local equilibrium *s* ≈ 7.2 × 10^−4^*k*_B_. Because superfluidity in the contacts enhances *I*_*N*_ while only slightly suppressing s^*^, superfluidity increases *I*_*S*_.

The fact that s^*^ > 0 directly shows that the large, non-Ohmic current between the two superfluids is itself not a pure supercurrent in the context of a two-fluid model^39^. The observation that the flow is resistive is insufficient alone to conclude that it is not superfluid as there are many mechanisms for resistance to arise in a pure supercurrent^40,41^. The observation that s^*^ ≫ *s* suggests that the channel is far from equilibrium and hydrodynamics breaks down as is often the case in weak link geometries^39^ unlike previous assumptions^31,38^. We discuss in Supplementary Information section 1 how the degree to which hydrodynamics breaks down depends on the preparation of the system. The large entropy current suggests an irreversible conversion process from superfluid currents in the contacts to normal currents in the channel and back to superfluid, or the propagation of normal currents originating in the normal regions of the reservoirs through the superfluids while remaining normal. Moreover, the independence of s^*^ from *ν*_*x*_ implies that this process is independent of the channel geometry. There is an analogy between this observation and the central result of Landauer–Büttiker theory that the conductance through a ballistic channel is also independent of the geometry and depends only on the channel’s transmission and the number of propagating modes.

**Fig. 3:**
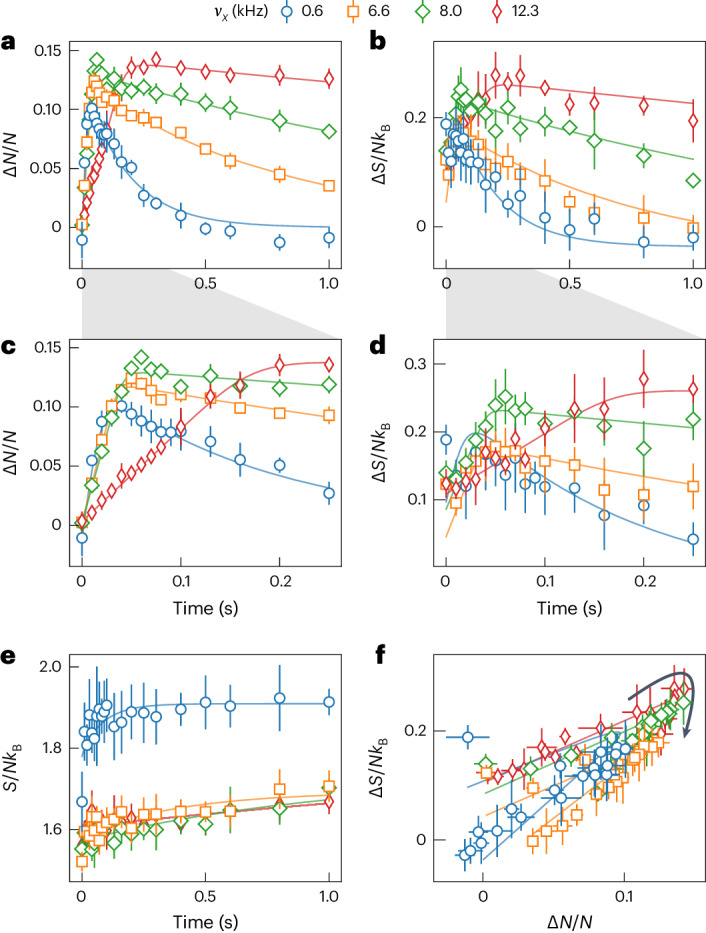
Competition between two modes of entropy transport: nonlinear advection and linear diffusion. **a**,**b**, A pure entropy imbalance induces currents of particles (**a**) and entropy (**b**) which are dominated by the nonlinear advective mode at early time and the linear diffusive mode at long times. For strong confinement (red) the system reaches a non-equilibrium steady state which persists beyond the experimentally accessible time, indicating the decoupling of advective and diffusive entropy transport. Reducing the confinement facilitates the diffusive mode, allowing equilibration and reducing the maximal response. **c**,**d**, Enlarged views of the initial dynamics of **a**,**b** for additional clarity. **e**, As in Fig. 2c, the increase in net entropy indicates the irreversibility of the transport process. A fit with our phenomenological model (solid lines) describes the data well over the explored parameter regime. **f**, The competition between the two modes, which changes with *ν*_*x*_, determines the path traced through state space. Error bars (data points) indicate the standard deviation (average) of three to five repetitions. Source data

In a second experiment, whose results are presented in Fig. 3, we prepare the system with a nearly pure entropy imbalance (Δ*N*(0) ≈ 0, Δ*S*(0) ≠ 0, open circle in Fig. 1b). The initial response of Δ*N* and Δ*S* from *t* = 0 to when Δ*N* reaches its maximum value is clearly non-exponential, resembling the advective dynamics in the first experiment with the same s^*^, while the dynamics that follow are much slower and consistent with exponential relaxation and therefore linear response. With decreasing *ν*_*x*_, both dynamics become faster and the maximum values of Δ*N* and Δ*S* achieved at the turning point become smaller. For the largest value of *ν*_*x*_, the initial response is still fast while the relaxation that follows is extremely slow and resembles a non-equilibrium steady state over experimentally accessible timescales: the relaxation time of this state is 8(2) s while it is reached from the initial state in only ~0.2 s. In this state, the non-vanishing imbalances Δ*N* and Δ*S* depend on the initial state as well as the path of the system through state space determined by s^*^, that is, the system is non-ergodic. This indicates that the non-equilibrium steady state previously observed at higher temperatures^30^ persists in the superfluid regime where the current with which it is reached is ≳6 times larger and non-Ohmic. Figure 3f shows the measured path in state space also illustrated in Fig. 1b. It demonstrates that the path is determined by the competition between the nonlinear and linear dynamics and varies with *ν*_*x*_, in contrast to the first experiment (Fig. 2d).

In the following, we formulate a minimal phenomenological model to describe our observations which are not captured by the linear response approach that successfully describes this system in the normal state^30,31^ as it predicts purely exponential relaxation. We therefore turn to the formalism of generalized gradient dynamics^42^, a generalization of Onsager’s theory of irreversible processes (Methods and Supplementary Information section 2). While it does not provide a microscopic theory, this formalism can describe general, irreversible, non-equilibrium processes and provides a convenient way to impose macroscopic constraints such as the second law of thermodynamics and conservation laws for the particle number and energy. Within this framework, we make the Ansatz

1 $$\begin{array}{rcl}{I}_{N}&=&{I}_{{{{\rm{exc}}}}}\tanh \left(\frac{\Delta \mu +{\alpha }_{\rm{c}}\Delta T}{\sigma }\right)\\ {I}_{S}&=&{\alpha }_{\rm{c}}{I}_{N}+{G}_{\rm{T}}\Delta T/T\end{array}$$

which produce entropy via the irreversible flow d*S*/d*t* = (*I*_*N*_Δ*μ* + *I*_*S*_Δ*T*)/*T*. The non-trivial result that *α*_c_ appears in both *I*_*N*_ and *I*_*S*_ is a generalization of Onsager’s reciprocal relations to nonlinear response and is a consequence of the irreversibility of these currents. The system exhibits two modes of entropy transport: a nonlinear advective mode ${I}_{S}^{\,\rm{a}}={\alpha }_{\rm{c}}{I}_{N}$ characterized by the excess current *I*_exc_, Seebeck coefficient *α*_c_ and nonlinearity *σ*, wherein each transported particle carries entropy s^*^ = *α*_c_ on average, and a linear diffusive mode ${I}_{S}^{\,\rm{d}}={G}_{\rm{T}}\Delta T/T$ characterized by the thermal conductance *G*_T_ which enables entropy transport without net particle transport according to Fourier’s law. The linear model is reproduced in the limit of large *σ* with conductance *G* = *I*_exc_/*σ*. In a Fermi liquid, these two modes are related by the Wiedemann–Franz law where the Lorenz number *L* = *G*_T_/*TG* has the universal value ${\uppi }^{2}{k}_{\rm {B}}^{2}/3$. The nonlinearity implies the breakdown of the Wiedemann–Franz law since the advective and diffusive modes are no longer linked^30^. The excess current *I*_exc_ is the particle current with the nonlinearity saturated, as in superconducting weak links^40^.

**Fig. 4:**
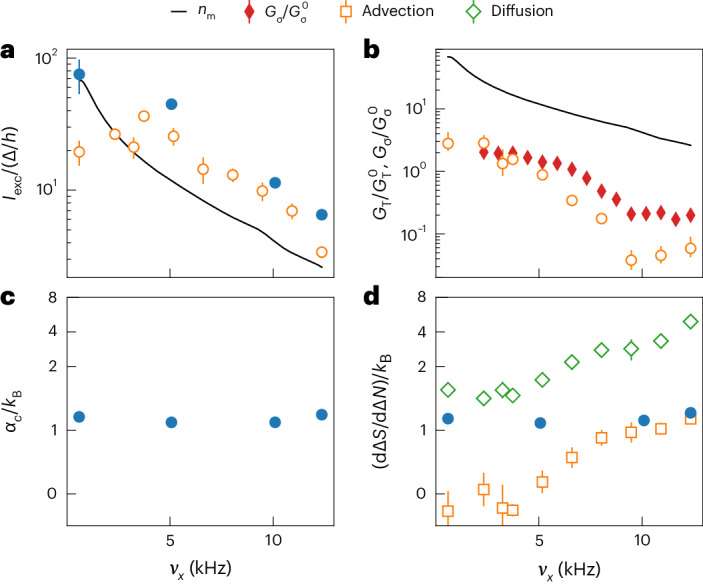
Characterizing the linear and nonlinear transport modes by fitting the phenomenological model across the 1D–2D crossover. Fitted parameters from the first (second) experiment corresponding to Fig. 2 (Fig. 3) are plotted with filled (open) markers here. **a**, The excess current normalized to the superfluid gap. **b**, The thermal and spin conductances normalized to the non-interacting values ${G}_{\rm{T}}^{0}=2{\uppi }^{2}{k}_{\rm {B}}^{2}T/3h$, ${G}_{\sigma }^{0}=2/h$ for a single transverse mode (see main text). For reference, the number of occupied transverse modes in the channel *n*_m_ is also shown in **a** and **b**. **c**, The Seebeck coefficient (advectively transported entropy per particle) extracted directly from the fits to the first experiment. **d**, The slope of the path through state space in the first experiment and in the advective (boxes) and diffusive (diamonds) limits of the second experiment. Error bars represent standard errors from the fits. Source data

Figure 4 shows the parameters of the model as functions of *ν*_*x*_ extracted from the fits shown as curves in Figs. 2 and 3. Panel a shows *I*_exc_ normalized by the fermionic superfluid gap *Δ*/*h* along with the number of occupied transverse modes at equilibrium *n*_m_ (Methods). Filled (open) circles were extracted from the first (second) experiment. *I*_exc_ follows *n*_m_*Δ*/*h*, increasing as *ν*_*x*_ decreases until *ν*_*x*_ ≈ 4 kHz where it plateaus, likely due to additional resistances in series with the 1D region. The fitted *I*_exc_ is apparently reduced in the second experiment relative to the first because the initial current is suppressed by the diffusive mode, making it more difficult to fit. It is intriguing that, consistent with previous studies^28,29^, the equilibrium superfluid gap *Δ* within the local density approximation is still the relevant scale for the current, despite the evidence that the channel region is far from equilibrium.

Figure 4b shows *G*_T_ and the spin conductance *G*_σ_ separately measured in the same system by preparing a pure spin imbalance (Methods) normalized to their values for the single-mode non-interacting ballistic quantum point contact ${G}_{\rm{T}}^{0}=2{\uppi }^{2}{k}_{\rm{B}}^{2}T/3h,\,{G}_{\sigma }^{0}=2/h$. Both conductances are suppressed relative to the non-interacting values and increase monotonically with decreasing *ν*_*x*_, possibly due to the appearance of non-degenerate transverse modes at the edges of the channel (Methods and Supplementary Information section 3). The non-equilibrium steady state arises from the fact that *G*_T_ → 0 in the 1D limit. The relative increase of *G*_T_ with decreasing *ν*_*x*_ is larger than that of *G*_σ_, suggesting that more types of excitations can contribute to diffusive entropy transport than spin transport, for example, both collective phonon and quasiparticle excitations can contribute to *G*_T_ (ref. ^43^) while only quasiparticle excitations can contribute to *G*_σ_ (ref. ^44^).

Figure 4c shows the fitted Seebeck coefficient *α*_c_, while Fig. 4d shows the slope of the path through state space dΔ*S*/dΔ*N* during the advective and diffusive dynamics. The fitted *α*_c_ and dΔ*S*/dΔ*N* match for the purely advective transport in the first experiment, showing that *α*_c_ is remarkably insensitive to *ν*_*x*_, while dΔ*S*/dΔ*N* more clearly shows how the two modes compete in the second experiment to determine the net response of the system. Figure 4d shows that, while both modes are generally present in the system’s dynamics, their relative prevalence depends on *ν*_*x*_ as well as the initial state: the initial state in the first experiment was carefully chosen to allow the system to relax to equilibrium via the advective mode alone by preparing Δ*S*(0) = *α*_c_Δ*N*(0) while the initial state in the second was chosen to contain both modes.

In summary, we have observed that the conceptually simple system of two superfluids connected by a ballistic channel exhibits the highly non-intuitive and currently unexplained effect that the presence of superfluidity increases the rate of irreversible entropy transport between them via nonlinear advection. This contrasts with the more familiar case of superfluid and superconducting tunnel junctions where the reversible, entropy-free Josephson current dominates. The entropy advectively carried per particle is nearly independent of the channel’s geometry, while the timescales of advective and diffusive transport depends strongly thereon, raising the question of the microscopic origin of the observed entropy transported per particle s^*^ ≃ 1*k*_B_. Our phenomenological model that captures these observations, in particular the identification of advective ${I}_{S}^{\,\mathrm{a}}\propto {I}_{N}$ and diffusive ${I}_{S}^{\,\mathrm{d}}\propto \Delta T$ modes along with the sigmoidal shape of *I*_*N*_(Δ*μ* + *α*_c_Δ*T*), may help guide future microscopic theories of the system. While extensive research has been conducted on the entropy producing effects of topological excitations of the superfluid order parameter^8,12,21,39,41,45–49^, less attention has been given to their influence on entropy transport and the possible pair-breaking processes they can induce. Early studies of superconductors found that mobile vortices can advectively transport entropy by carrying pockets of normal fluid^50^ with them^24,51–53^. More generally, entropy-carrying topological excitations, which give rise to a finite chemical potential bias according to the Josephson relation Δ*μ* = *hN*_v_/d*t* (refs. ^39^), where *N*_v_ is the number of vortices, can result from a complex spatial structure of the order parameter^49^. Alternatively, it is possible that an extension of microscopic theories of multiple Andreev reflection^37,54^, which reproduce the finding that the excess current scales linearly with the number of channels and the gap^28,29^, may explain our observations. Clearly, a proper microscopic theory of this system is a challenge for the future. A complete understanding of the particle and entropy transport in superfluid systems is essential for both fundamental and technological purposes.

## Methods

### Transport configuration

The atoms are trapped magnetically along *y* and optically by a red-detuned beam along *x* and *z*, with confinement frequencies *ν*_trap,*x*_ = 171(1) Hz, *ν*_trap,*y*_ = 28.31(2) Hz and *ν*_trap,*z*_ = 164(1) Hz. A pair of repulsive TEM_01_-like beams propagating along *x* and *z*, which we call the lightsheet (LS) and wire respectively, intersect at the centre of the trapped cloud and separate it into two reservoirs connected by a channel. The transverse confinement frequencies at the centre are set to *ν*_*z*_ = 9.42(6) kHz (*k*_B_*T*/*hν*_*z*_ = 0.21) and *ν*_*x*_ = 0.61…12.4(2) kHz (*k*_B_*T*/*hν*_*x*_ = 0.16…3.3) with the powers of the beams. An attractive Gaussian beam propagating along *z* with a similar size as the LS beam acts as a gate potential in the channel. The peak gate potential is ${V}_{{{{\rm{gate}}}}}^{\;0}=-2.17(1)\,\upmu {{{\rm{K}}}}\times {k}_{\rm{B}}$. We use a wall beam, which is thin along *y* and wide along *x*, during preparation and imaging to completely block transport with a barrier height ${V}_{{{{\rm{wall}}}}}^{\;0}$ much larger than *μ* and *k*_B_*T*. The repulsive LS, wire and wall are generated using blue-detuned 532 nm light while the attractive gate is created with red-detuned 766.7 nm light.

The effective potential energy landscape along *y* at *x* = *z* = 0 (Supplementary Information section 3) is approximately *V*_eff_(*y*, *n*_*x*_, *n*_*z*_) ≈ *hν*_*x*_(*y*)(*n*_*x*_ + 1/2) + *hν*_*z*_(*y*)(*n*_*z*_ + 1/2), where *ν*_*x*_ and *ν*_*z*_ vary along *y* due to the beams’ profile and *n*_*x*_, *n*_*z*_ are the quantum numbers of the harmonic potential in *x* and *z* directions. The number of occupied transverse modes *n*_m_ (Fig. 4) is calculated via the Fermi–Dirac occupation with local chemical potential set by *V*_eff_(*y*, *n*_*x*_, *n*_*z*_),

2 $${n}_{\rm{m}}=\mathop{\sum }\limits_{{n}_{x},{n}_{z}=0}^{\infty }\mathop{\min }\limits_{y}\frac{1}{1+\exp \left\{[{V}_{{{{\rm{eff}}}}}(\;y,{n}_{x},{n}_{z})-\mu ]/{k}_{\rm{B}}T\right\}}$$

where the minimum occupation of each mode is used to account for modes that are not always occupied throughout the channel (Supplementary Information section 3).

The complete potential energy landscape *V*(**r**), where **r** = (*x*, *y*, *z*), was used to produce Fig. 1a via the local density approximation for the density *n*(**r**) = *n*[*μ* − *V*(**r**), *T*] (refs. ^11,36^) that determines the local Fermi temperature ${k}_{\rm{B}}{T}_{\rm{F}}({{{\bf{r}}}})={\hslash }^{2}{[3{\uppi }^{2}n({{{\bf{r}}}})]}^{2/3}/2m$, where *m* is the atomic mass. The superfluid gap *Δ* assuming local equilibrium is estimated using the calculation in a homogeneous system *Δ*(*μ*_c_, *T*) (ref. ^55^) where ${\mu }_{\rm{c}}=\mathop{\max }\limits_{{{{\bf{r}}}}}[\mu -V({{{\bf{r}}}})]$ is the maximum local chemical potential in the system. The crossover between 1D and 2D regimes (*ν*_*x*_ ≈ 7 kHz) of the channel is estimated by comparing the local degeneracy along *x* (at *y* = *z* = 0) to the superfluid transition. In the 2D limit, non-superfluid modes can pass through the edges of the channel while in the 1D limit the degeneracy across the channel is below the superfluid transition (Supplementary Information section 3).

### Transport preparation

To prepare imbalances Δ*S*(0), Δ*N*(0) we ramp up the channel beams to separate the two reservoirs followed by forced optical evaporation. Using a magnetic field gradient along *y*, we shift the centre of the magnetic trap with respect to the channel beams before separation to prepare Δ*N*(0). By shifting the trap centre during evaporation, we can compress one reservoir and decompress the other, thereby changing their evaporation efficiencies and inducing a controllable Δ*S*(0). See Supplementary Information Section 4 for more details. To measure the spin conductance *G*_σ_ (Fig. 4b), we prepare a ‘magnetization’ imbalance Δ*M* = Δ*N*_*↑*_ − Δ*N*_*↓*_. To do this, we ramp down the magnetic field before separating the reservoirs at 52 G where the spins’ magnetic moments are different and modulate a magnetic gradient along *y* until the two spins oscillate out of phase. We then separate the two reservoirs and ramp back the magnetic field.

### Imaging and thermometry

Between the end of transport and the start of imaging, we ramp down the channel beams while keeping the wall on. At the end of each run, we obtain the column density ${n}_{i\sigma }^{{{{\rm{col}}}}}(\;y,z)$ of both reservoirs *i* = L, R and both spin states *σ* = *↓*, *↑* (first and third-lowest states in the ground state manifold) from two absorption images taken in quick succession in situ. We fit the degeneracy *q*_*iσ*_ = *μ*_*iσ*_/*k*_B_*T*_*iσ*_ and temperature *T*_*iσ*_ of both reservoirs for each spin state using the EoS of the harmonically trapped gas^11^. However, we use the fitted temperature from the first image (*↓*) for both spins since the density distribution in the second image is slightly perturbed by the first imaging pulse. The thermometry is calibrated using the critical *S*/*N* of the condensation phase transition on the BEC side of the Feshbach resonance. See Supplementary Information section 5B for more details.

### Generalized gradient dynamics

To ensure that the phenomenological nonlinear model satisfies basic properties such as the second law of thermodynamics, it is formulated it in terms of a dissipation potential *Ξ* (ref. ^42^). The thermodynamic fluxes are defined as derivatives of the dissipation potential *Ξ* with respect to the forces *I*_*N*_ = *T*∂*Ξ*/∂Δ*μ* and *I*_*S*_ = *T*∂*Ξ*/∂Δ*T*. In this formalism, Onsager reciprocity and the conservation of particles and energy are fulfilled. Our model (equation (1)) is the result of the following dissipation potential, which is constructed based on the experimental observation *I*_*S*_ = s^*^I_*N*_ and that *I*_*N*_ follows a sigmoidal function of Δ*μ* (ref. ^28^),

3 $$\varXi =\frac{\sigma {I}_{{{{\rm{exc}}}}}}{T}\log \left[\cosh \left(\frac{\Delta \mu +{s}^{* }\Delta T}{\sigma }\right)\right]+\frac{{G}_{\rm{T}}}{2}{\left(\frac{\Delta T}{T}\right)}^{2}$$

where the first part describes the advective and the second part the diffusive transport mode. See Supplementary Information section 2 for more details.

### Reservoir thermodynamics

To formulate equations of motion in state space (Δ*N*, Δ*S*), we relate Δ*μ*, Δ*T* to Δ*N*, Δ*S* in terms of thermodynamic response functions

4 $$\left(\begin{array}{c}\Delta N\\ \Delta S\end{array}\right)\approx \frac{\kappa }{2}\left(\begin{array}{cc}1&{\alpha }_{\rm{r}}\\ {\alpha }_{\rm{r}}&{\ell }_{\rm{r}}+{\alpha }_{\rm{r}}^{2}\end{array}\right)\left(\begin{array}{c}\Delta \mu \\ \Delta T\end{array}\right)$$

where *κ* is the compressibility, *α*_r_ is the dilatation coefficient and *ℓ*_r_ is the ‘Lorenz number’ of the reservoirs^56^ (Supplementary Information section 5A). To obtain the spin conductance, we use Δ*M* = (*χ*/2)Δ*b*, where Δ*b* = (Δ*μ*_*↑*_ − Δ*μ*_*↓*_)/2. The spin susceptibility *χ* ≈ 0.32*κ*, following the computed EoS of a polarized unitary Fermi gas^57^.

The potential landscape *V*(**r**) during the transport experiment deviates from simple harmonic potential due to the confinement beams as well as the anharmonicity of the optical dipole trap. We estimate from numeric simulations based on our knowledge of the *V*(**r**) that *T* and *κ* agree within 1% to those determined from absorption imaging in near-harmonic traps while *μ* is 24% higher during transport. However, *α*_r_ and *ℓ*_r_ are more sensitive to the trap potential and can deviate by a factor of 3. We therefore fit these response coefficients in our model. See Supplementary Information section 5A for more details.

The total entropy *S*, being a state variable (contour plot in Fig. 1b), depends on the imbalances Δ*N*(*t*) and Δ*S*(*t*) but not the currents *I*_*N*_ and *I*_*S*_. With the linearized reservoir response, the entropy produced by equilibration is given by

5 $$S(t)\approx {S}_{{{{\rm{eq}}}}}-\frac{\Delta {N}^{2}(t)}{2T\kappa }-\frac{{[\Delta S(t)-{\alpha }_{\rm{r}}\Delta N(t)]}^{2}}{2T{\ell }_{\rm{r}}\kappa }$$

where *S*_eq_ is the maximum entropy at equilibrium given fixed total *N* and *U*. The increase in *S*/*Nk*_B_ with decreasing *ν*_*x*_ in Figs. 2c and 3c is caused by switching on the wall beam to block transport. For lower *ν*_*x*_, there are more atoms in the channel to be perturbed by this process.

### Fitting procedure

We fit the phenomenological model (equation (1)) with linear reservoir responses (equation (4)) to each dataset—the set of different transport times at fixed *ν*_*x*_—independently for both the first and second experiment. We do this by solving the initial value problem for Δ*N*(*t*) and Δ*S*(*t*) given the parameters *α*_c_, *I*_exc_, *σ* and *G*_T_ along with the reservoir response functions *κ*, *α*_r_ and *ℓ*_r_. From these solutions, we also compute the total entropy *S*(*t*) as a function of time (equation (5)). We fit Δ*N*(*t*), Δ*S*(*t*) and *S*(*t*) simultaneously to the data using a least-squares fit. For the first experiment, only *I*_exc_, *σ*, *α*_c_, Δ*S*(0) and *S*_eq_ are free parameters. Other parameters are fixed to their theoretical values for better fit stability. For the second experiment, we fit *σ*, Δ*S*(0), *S*_eq_, *G*_T_, *α*_r_, *ℓ*_r_ and an offset in Δ*S* to account for drifts in alignment. *α*_c_ is fixed to the averaged value obtained in the first experiment. See Supplementary Information section 6 for more details. The slopes shown in Fig. 4d are obtained by simple linear fits in the state space Δ*S* versus Δ*N* (Figs. 2d and 3f). The advective and diffusive modes in the second experiment are separated in time at the maximum Δ*N*(*t*) (Fig. 2a).

## Online content

Any methods, additional references, Nature Portfolio reporting summaries, source data, extended data, supplementary information, acknowledgements, peer review information; details of author contributions and competing interests; and statements of data and code availability are available at 10.1038/s41567-024-02483-3.

### Supplementary information

Supplementary Information Supplementary Sections 1–6 and Figs. 1–4.

### Source data

Source Data Fig. 2 Statistical source data.

Source Data Fig. 3 Statistical source data.

Source Data Fig. 4 Statistical source data.

## Data Availability

All data files are available from the corresponding authors upon reasonable request. Source data are provided with this paper.

## Code availability

Source codes for the data processing and numerical simulations are available from the corresponding authors upon reasonable request.

## References

[1] Senior J (2020). Heat rectification via a superconducting artificial atom. Commun. Phys..

[2] Potel G, Barranco F, Vigezzi E, Broglia RA (2021). Quantum entanglement in nuclear Cooper-pair tunneling with *γ* rays. Phys. Rev. C..

[3] Shelly CD, Matrozova EA, Petrashov VT (2016). Resolving thermoelectric ‘paradox’ in superconductors. Sci. Adv..

[4] Fornieri A, Giazotto F (2017). Towards phase-coherent caloritronics in superconducting circuits. Nat. Nanotechnol..

[5] Ginzburg VL (1989). Thermoelectric effects in superconductors. J. Supercond..

[6] Crossno J (2016). Observation of the Dirac fluid and the breakdown of the Wiedemann–Franz law in graphene. Science.

[7] Dogra LH (2023). Universal equation of state for wave turbulence in a quantum gas. Nature.

[8] Bradley DI (2011). Direct measurement of the energy dissipated by quantum turbulence. Nat. Phys..

[9] Pekola JP, Karimi B (2021). *Colloquium*: quantum heat transport in condensed matter systems. Rev. Mod. Phys..

[10] Ibabe A (2023). Joule spectroscopy of hybrid superconductor-semiconductor nanodevices. Nat. Commun..

[11] Ku MJH, Sommer AT, Cheuk LW, Zwierlein MW (2012). Revealing the superfluid lambda transition in the universal thermodynamics of a unitary Fermi gas. Science.

[12] Valtolina G (2015). Josephson effect in fermionic superfluids across the BEC-BCS crossover. Science.

[13] Luick N (2020). An ideal Josephson junction in an ultracold two-dimensional Fermi gas. Science.

[14] Patel PB (2020). Universal sound diffusion in a strongly interacting Fermi gas. Science.

[15] Wang X, Li X, Arakelyan I, Thomas JE (2022). Hydrodynamic relaxation in a strongly interacting Fermi gas. Phys. Rev. Lett..

[16] Sidorenkov LA (2013). Second sound and the superfluid fraction in a Fermi gas with resonant interactions. Nature.

[17] Li X (2022). Second sound attenuation near quantum criticality. Science.

[18] Yan Z (2024). Thermography of the superfluid transition in a strongly interacting Fermi gas. Science.

[19] Del Pace G, Kwon WJ, Zaccanti M, Roati G, Scazza F (2021). Tunneling transport of unitary fermions across the superfluid transition. Phys. Rev. Lett..

[20] Agraït N, Yeyati AL, Ruitenbeek JM (2003). Quantum properties of atomic-sized conductors. Phys. Rep..

[21] Hoskinson E, Sato Y, Hahn I, Packard RE (2006). Transition from phase slips to the Josephson effect in a superfluid ^4^He weak link. Nat. Phys..

[22] Botimer J, Taborek P (2016). Pressure driven flow of superfluid ^4^He through a nanopipe. Phys. Rev. Fluids.

[23] Krinner S, Stadler D, Husmann D, Brantut J-P, Esslinger T (2014). Observation of quantized conductance in neutral matter. Nature.

[24] Tinkham, M. *Introduction to Superconductivity* 2nd edn (McGraw Hill, 1996).

[25] Chen Y, Lin Y-H, Snyder SD, Goldman AM, Kamenev A (2014). Dissipative superconducting state of non-equilibrium nanowires. Nat. Phys..

[26] Viljas JK (2005). Multiple Andreev reflections in weak links of superfluid ^3^He−*B*. Phys. Rev. B.

[27] Stadler D, Krinner S, Meineke J, Brantut J-P, Esslinger T (2012). Observing the drop of resistance in the flow of a superfluid Fermi gas. Nature.

[28] Husmann D (2015). Connecting strongly correlated superfluids by a quantum point contact. Science.

[29] Huang M-Z (2023). Superfluid signatures in a dissipative quantum point contact. Phys. Rev. Lett..

[30] Husmann D (2018). Breakdown of the Wiedemann-Franz law in a unitary Fermi gas. Proc. Natl Acad. Sci. USA.

[31] Häusler S (2021). Interaction-assisted reversal of thermopower with ultracold atoms. Phys. Rev. X.

[32] Breuer, H.-P. & Petruccione, F. *The Theory of Open Quantum Systems* (Oxford Univ. Press, 2002).

[33] Esposito M, Lindenberg K, Broeck CVD (2010). Entropy production as correlation between system and reservoir. New J. Phys..

[34] Kaufman AM (2016). Quantum thermalization through entanglement in an isolated many-body system. Science.

[35] Gnezdilov NV, Pavlov AI, Ohanesjan V, Cheipesh Y, Schalm K (2023). Ultrafast dynamics of cold Fermi gas after a local quench. Phys. Rev. A.

[36] Haussmann R, Zwerger W (2008). Thermodynamics of a trapped unitary Fermi gas. Phys. Rev. A.

[37] Yao J, Liu B, Sun M, Zhai H (2018). Controlled transport between Fermi superfluids through a quantum point contact. Phys. Rev. A.

[38] Kanász-Nagy M, Glazman L, Esslinger T, Demler EA (2016). Anomalous conductances in an ultracold quantum wire. Phys. Rev. Lett..

[39] Varoquaux E (2015). Anderson’s considerations on the flow of superfluid helium: some offshoots. Rev. Mod. Phys..

[40] Likharev KK (1979). Superconducting weak links. Rev. Mod. Phys..

[41] Halperin BI, Refael G, Demler E (2010). Resistance in superconductors. Int. J. Mod. Phys. B.

[42] Pavelka, M., Klika, V. & Grmela, M. *Multiscale Thermo-Dynamics: Introduction to GENERIC* (De Gruyter, 2018).

[43] Uchino S (2020). Role of Nambu–Goldstone modes in the fermionic-superfluid point contact. Phys. Rev. Res..

[44] Sekino Y, Tajima H, Uchino S (2020). Mesoscopic spin transport between strongly interacting Fermi gases. Phys. Rev. Res..

[45] Silaev MA (2012). Universal mechanism of dissipation in Fermi superfluids at ultralow temperatures. Phys. Rev. Lett..

[46] Barenghi CF, Skrbek L, Sreenivasan KR (2014). Introduction to quantum turbulence. Proc. Natl Acad. Sci. USA.

[47] D’Errico C, Abbate SS, Modugno G (2017). Quantum phase slips: from condensed matter to ultracold quantum gases. Philos. Trans. R. Soc. A.

[48] Burchianti A (2018). Connecting dissipation and phase slips in a Josephson junction between fermionic superfluids. Phys. Rev. Lett..

[49] Wlazlowski G, Xhani K, Tylutki M, Proukakis NP, Magierski P (2023). Dissipation mechanisms in fermionic Josephson junction. Phys. Rev. Lett..

[50] Sensarma R, Randeria M, Ho T-L (2006). Vortices in superfluid Fermi gases through the BEC to BCS crossover. Phys. Rev. Lett..

[51] Solomon PR, Otter FA (1967). Thermomagnetic effects in superconductors. Phys. Rev..

[52] Solomon PR (1969). Flux motion in type-I superconductors. Phys. Rev..

[53] Vidal F (1973). Low-frequency ac measurements of the entropy flux associated with the moving vortex lines in a low-*κ* type-II superconductor. Phys. Rev. B.

[54] Setiawan F, Hofmann J (2022). Analytic approach to transport in superconducting junctions with arbitrary carrier density. Phys. Rev. Res..

[55] Haussmann R, Rantner W, Cerrito S, Zwerger W (2007). Thermodynamics of the BCS–BEC crossover. Phys. Rev. A.

[56] Grenier C, Kollath C, Georges A (2016). Thermoelectric transport and Peltier cooling of cold atomic gases. C. R. Phys..

[57] Rammelmüller L, Loheac AC, Drut JE, Braun J (2018). Finite-temperature equation of state of polarized fermions at unitarity. Phys. Rev. Lett..

