# Supplementary material for: Irreversible entropy transport enhanced by fermionic superfluidity
Source: Nat Phys. 2024 Apr 22;20(7):1091–6. doi: 10.1038/s41567-024-02483-3 (PMC11254751; doi:10.1038/s41567-024-02483-3)
Supplement: Supplementary file 1 — Supplementary Sections 1–6 and Figs. 1–4. [file 41567_2024_2483_MOESM1_ESM.pdf]

nature physics

Article https://doi.org/10.1038/s41567-024-02483-3

Irreversible entropy transport enhanced by fermionic superfluidity

In the format provided by the authors and unedited

Supplementary Information

1. BREAKDOWN OF HYDRODYNAMICS

The two-fluid model states that the total particle current is the sum of the normal and superfluid (Josephson) components $I_N = I_N^n + I_N^s$ while the entropy current is carried advectively by only the normal current $I_S = s_n^* I_N^n$. We observe that the entropy current is proportional to the total particle current $I_S = s^* I_N$ which, when combined with the two previous equations, yields $I_N^s = (s_n^*/s^* - 1) I_N^n$. The normal particle current I_N^n is given by the normal particle current density j_n integrated over the cross section at the centre of the channel and the entropy current is given by j_n weighted by the local entropy per particle

$$\begin{aligned} I_N^n &= \int \mathrm{d}x\,\mathrm{d}z\, j_n(x, y=0, z) \\ I_S &= \int \mathrm{d}x\,\mathrm{d}z\, j_n(x, y=0, z) s(x, y=0, z). \end{aligned} \tag{S1}$$

If the system is hydrodynamic, the local entropy per particle is given by its value near equilibrium [1, 2], so the entropy transported per particle by the normal current would therefore be $s_n^* = I_S/I_N^n \sim s \approx 7.2 \times 10^{-4} k_B$. This is several orders of magnitude smaller than the observed entropy transported per particle $s^* = 1.18(3) k_B$, implying that $I_N^s \approx -I_N^n$, i.e., the normal and superfluid currents flow in opposite directions and their small difference is the observed net current. Depending on whether or not the current-phase relation is multi-valued [3], the time-averaged supercurrent at finite $\Delta\mu$ either vanishes by undergoing reversible, adiabatic phase slips (AC Josephson effect) [4, 5] or flows in the same direction as the normal current by irreversibly nucleating topological excitations such as vortices [6]. A simple picture of this process is that the force $\propto -\Delta\mu$ accelerates the superfluid until it reaches its critical velocity, at which point it converts some kinetic energy to heat, then begins to accelerate again and the process repeats [7]. This contradiction–that our observations imply $I_N^s \approx -I_N^n$ if the system is hydrodynamic while the equations of motion for the superfluid require $\mathrm{sign}(I_N^s) = \mathrm{sign}(I_N^n)$–shows that the hydrodynamic two-fluid model, namely that the transported entropy is determined by the near-equilibrium local entropy in the contacts, is not valid in the channel despite the internal equilibrium of the reservoirs sufficiently far from the channel (Sec. 2). This argument does not rely on the precise values of s and s^*, just on the fact that $s < s^*$. This means that the finding that hydrodynamic theory breaks down is robust to changes in s^* over approximately three orders of magnitude.

As shown in the main text, superfluidity in the reservoirs increased the speed of entropy transport between them. In a bulk hydrodynamic system, entropy transport is faster in a superfluid, where entropy propagates as waves of second sound [8], than in a normal fluid where it propagates diffusively [9]. Because it is a wave, second sound is in principle a reversible mechanism of particle and entropy transport, though the damping that often accompanies it [10] could allow it to transport entropy irreversibly as we observe here. Because the gas in the channel is far from equilibrium, second sound in a strict hydrodynamic sense [11] cannot propagate through it. Nevertheless, second sound in a more general, far-from-equilibrium sense referring to any relative motion between the normal and superfluid components induced by fundamental excitations [12] may be present.

The channel and wall beams (Sec. 3) are on during the preparation of the system (Sec. 4), while the gate beam is ramped on over 10 ms followed by 5 ms of settling before the wall is switched off to allow transport. We have verified that our observations of the advective mode, quantified by the values of I_{exc}, σ, and s^*, remain unchanged for settling times up to 0.5 s after the gate beam ramp time, much longer than the advective relaxation time (cf. Fig. 1). Longer

settling times up to 3.5 s lead to slower, more exponential relaxation of ΔN (smaller I_{exc} and larger σ), though the effect is consistent with heating induced by the gate beam. Nevertheless, using a high-resolution imaging system focused on the channel, we observe that the density in the region addressed by the gate previously referred to as pockets [13] increases very slowly after the ramp on a timescale of several seconds, confirming the non-equilibrium nature of the channel. The fact that the observed nonlinear dynamics are much faster than the local equilibration time of the channel and remain unchanged over a large range of preparation times suggests that the far-from-equilibrium state of the channel is robust and well-defined by the equilibrium reservoirs and channel geometry.

2. GENERALISED GRADIENT DYNAMICS

To ensure that our phenomenological nonlinear model satisfies some basic properties such as the Second Law of Thermodynamics and the conservation of particles and energy, it is useful to formulate it in terms of a dissipation potential Ξ [14, 15]. Both Onsager's theory of irreversible processes [16] and the more general formalism of gradient dynamics [15] identify thermodynamic forces and fluxes which determine the entropy production rate of a system $\dot{S} = \mathrm{d}S/\mathrm{d}t$. This production rate $\dot{S}$ can be written in terms of thermodynamic observables from the first law of thermodynamics $\mathrm{d}S = \sum_i x_i \, \mathrm{d}X_i$ which relates the change in a system's entropy due to changes in the extensive quantities that characterise the system X_i via their conjugate intensive variables x_i [16], e.g., chemical potential, temperature, s-wave scattering length, normal and superfluid velocities, trap frequency, channel beam powers, etc. For a spin-balanced gas at unitarity in a static trap, this is

$$\mathrm{d}S = \frac{1}{T}\,\mathrm{d}U - \frac{\mu}{T}\,\mathrm{d}N\,. \tag{S2}$$

By dividing both sides of the equation by the time differential $\mathrm{d}t$, we find the entropy production rate

$$\dot{S} = \frac{\mathrm{d}S}{\mathrm{d}t} = \frac{1}{T}\frac{\mathrm{d}U}{\mathrm{d}t} - \frac{\mu}{T}\frac{\mathrm{d}N}{\mathrm{d}t} \tag{S3}$$

which has the interpretation that if energy and atoms are added/removed at rates $\dot{U}$ and $\dot{N}$ while μ and T are held constant, then the entropy increases/decreases at a rate $\dot{S}$ due to the thermalization of non-equilibrium excitations produced by $\dot{U}$ and $\dot{N}$. Since the total entropy of the system is the sum of the entropies of the left and right reservoirs, the net entropy production rate

$$\dot{S} = \dot{S}_L + \dot{S}_R \tag{S4}$$

is given in terms of the production rates in the two reservoirs

$$\dot{S}_i = \frac{1}{T_i}\dot{U}_i - \frac{\mu_i}{T_i}\dot{N}_i. \tag{S5}$$

Because the total energy and atom number is conserved

$$\begin{aligned} \dot{U} &= \dot{U}_L + \dot{U}_R = 0 \\ \dot{N} &= \dot{N}_L + \dot{N}_R = 0, \end{aligned} \tag{S6}$$

their addition/removal rates in each reservoir are given by the conserved currents flowing between them

$$\begin{aligned} I_U &= -\frac{1}{2}(\dot{U}_L - \dot{U}_R) = -\frac{1}{2}\frac{\mathrm{d}\Delta U}{\mathrm{d}t} \\ I_N &= -\frac{1}{2}(\dot{N}_L - \dot{N}_R) = -\frac{1}{2}\frac{\mathrm{d}\Delta N}{\mathrm{d}t} \end{aligned} \tag{S7}$$

Combining this with Eq. S4 and S5, we find that $\dot{S}$ can be written equivalently in terms of either I_N and I_U or, without any approximations, I_N and the apparent entropy current $I_S = -(1/2)\,\mathrm{d}\Delta S/\mathrm{d}t$

$$\dot{S} = I_N \Delta\left(\frac{\mu}{T}\right) + I_U \Delta\left(-\frac{1}{T}\right) = \frac{I_N \Delta\mu + I_S \Delta T}{T} \tag{S8}$$

where $T = (T_L + T_R)/2$. This means we can formulate a theory with either the fluxes I_N and I_U driven by the forces $\Delta(\mu/T) = \mu_L/T_L - \mu_R/T_R$ and $\Delta(-1/T) = -1/T_L + 1/T_R$ or the fluxes I_N and I_S driven by the forces $\Delta\mu/T$ and $\Delta T/T$ or simply $\Delta\mu$ and ΔT. We chose the latter as the entropy is more natural to measure given our experimental sequence (the energy is not conserved by the channel beam ramps but entropy is) and because it gives deeper insight into the nature of the flow between the two reservoirs. The vector of state variables is therefore $\mathbf{X} = (N, U, \Delta N, \Delta S)$ and the vector of their conjugate quantities are $\mathbf{x} = \partial S/\partial \mathbf{X} = (-\mu/T, 1/T, -\Delta\mu/2T, -\Delta T/2T)$, though practically they can be reduced to $\mathbf{X} = (\Delta N, \Delta S)$ and $\mathbf{x} = (-\Delta\mu/2T, -\Delta T/2T)$ because $\dot{N} = \dot{U} = 0$. The energy and entropy currents are related by

$$I_U = \left(T - \frac{\Delta T^2}{4T}\right) I_S + \left(\mu - \frac{\Delta\mu \Delta T}{4T}\right) I_N \tag{S9}$$

so, for small biases $\Delta T \ll T, \Delta\mu \ll \mu$, the entropy current is approximately given by the heat current

$$I_Q = I_U - \mu I_N \approx T I_S. \tag{S10}$$

The fluxes are defined as derivatives of the dissipation potential Ξ with respect to the forces

$$I_N = \frac{\partial \Xi}{\partial \Delta(\mu/T)},\ I_U = \frac{\partial \Xi}{\partial \Delta(-1/T)} \quad \text{or} \quad I_N = T\frac{\partial \Xi}{\partial \Delta\mu},\ I_S = T\frac{\partial \Xi}{\partial \Delta T} \tag{S11}$$

Since Ξ is a single-valued scalar potential function of two scalars, Onsager reciprocity

$$\frac{\partial I_N}{\partial \Delta(-1/T)} = \frac{\partial I_U}{\partial \Delta(\mu/T)} \quad \text{or} \quad \frac{\partial I_N}{\partial \Delta T} = \frac{\partial I_S}{\partial \Delta\mu} \tag{S12}$$

is naturally fulfilled in this formalism at any $\Delta(\mu/T), \Delta(-1/T)$ or $\Delta\mu, \Delta T$, not just in the linear response regime near equilibrium as is usually the case when imposing this condition.

To formulate equations of motion for the evolution in state space $\Delta N(t), \Delta S(t)$, we need to relate $\Delta\mu, \Delta T$ to $\Delta N, \Delta S$ via the reservoirs' equation of state (EoS). For the relatively small imbalances used here, we can linearise the EoS in terms of thermodynamic response functions

$$\begin{pmatrix} \Delta N \\ \Delta S \end{pmatrix} \approx \frac{\kappa}{2} \begin{pmatrix} 1 & \alpha_r \\ \alpha_r & \ell_r + \alpha_r^2 \end{pmatrix} \begin{pmatrix} \Delta\mu \\ \Delta T \end{pmatrix} \tag{S13}$$

where κ is the compressibility, α_r is the dilatation coefficient, and ℓ_r is the "Lorenz number" of the reservoirs [17]; see Sec. 5 for definitions. Within the same approximation, the energy and

entropy imbalance are related by the average chemical potential $\mu = (\mu_L + \mu_R)/2$ and temperature $T = (T_L + T_R)/2$

$$\Delta U \approx \mu\Delta N + T\Delta S. \tag{S14}$$

This formalism obeys the principle that S is a state variable: S depends only on N, U, ΔN, and ΔS but not on the path the system travelled in state space. In other words, the entropy produced by equilibration is the entropy difference between the initial and final states

$$\begin{aligned}\delta S &= \int_0^\infty \mathrm{d}t\,\dot{S}(t) = \int_0^\infty \mathrm{d}t\,\frac{I_N(t)\Delta\mu(t) + I_S(t)\Delta T(t)}{T(t)}\\ &= \int_0^\infty \mathrm{d}t\,\dot{\mathbf{X}}\cdot\mathbf{x} = \int_{\mathbf{X}_0}^{\mathbf{X}_\infty} \mathrm{d}\mathbf{X}\cdot\frac{\partial\Xi}{\partial\mathbf{X}}\\ &= S(N,U,0,0) - S(N,U,\Delta N_0,\Delta S_0)\end{aligned} \tag{S15}$$

and is independent of the microscopic processes and current-bias characteristics of the system contained in Ξ. If we use the linearised reservoir response in Eq. S13, this becomes

$$\delta S \approx \frac{\Delta N_0^2}{2T\kappa} + \frac{(\Delta S_0 - \alpha_r\Delta N_0)^2}{2T\ell_r\kappa} \tag{S16}$$

which, together with $S(N,U,0,0)$, is the expression plotted in Fig. 1b. The interpretation of this result is that there is initially some potential energy in the system due to the imbalances, like the charging energy of a capacitor, which, due to the Joule heating from irreversible flow through the resistive channel, is converted into heat energy, i.e., entropy. We have verified that the system is nearly closed by ensuring the absence of particle loss $|\mathrm{d}N/\mathrm{d}t|/N < 0.01\,\mathrm{s}^{-1}$ and heating in equilibrium $\mathrm{d}(S/Nk_B)/\mathrm{d}t < 0.02\,\mathrm{s}^{-1}$ due to, e.g., photon scattering from the beams, vacuum background collisions, and three-body recombination [18]. The total duration of the sequence is kept constant, varying only the amount of time the channel is open, so the entropy production plotted in Fig. 2c and Fig. 3c includes the small and constant offset coming from residual heating not due to irreversible transport. The increase in S/Nk_B with decreasing ν_x in Fig. 2c and Fig. 3c is caused by switching on the wall beam to block transport. This process affects low ν_x more since there are many more atoms in the channel in that case to absorb the potential energy. This additional heat does not affect the state of the system during transport as it is only added afterwards.

This formalism with the state vector $\mathbf{X} = (N, U, \Delta N, \Delta S)$ only applies when the reservoirs are in internal equilibrium, i.e., the time evolution is quasi-stationary. If, for example, they were hydrodynamic and their breathing modes were excited, then the amplitudes and phases of these modes would have to be added to the state vector $\mathbf{X}$ to completely characterise the state of the system. Once the complete set of experimentally distinguishable degrees of freedom are added to $\mathbf{X}$, this formalism applies again even away from equilibrium. The fact that our observations are consistent with a description using only these four state variables of the reservoirs confirms the assumptions that the atom number and energy are conserved and that the reservoirs are in internal equilibrium during transport. It is therefore natural to ask whether this formalism still applies when the channel is far from equilibrium and in principle contains many degrees of freedom that must be added to $\mathbf{X}$. Indeed it does because the state of the channel is fixed by its boundary conditions imposed by the equilibrium reservoirs. Therefore, each additional degree of freedom of the channel is a function of only the four state variables in $\mathbf{X}$ and need not be added for a complete description.

Generalised gradient dynamics can only account for irreversible dynamics, however a phase bias $\Delta\phi$ between the superfluids can in principle drive a reversible Josephson supercurrent $I_N \sim I_c \sin(\Delta\phi)$ which transports and produces no entropy [19]. Our observations show that

the dynamics are irreversible in nature since the particle current both transports and produces entropy and we observe no undamped oscillations characteristic of the Josephson effect in a finite system [20, 21]. It is nevertheless somewhat surprising that we do not observe this effect, however this is likely a consequence of the high transmission of the channel. In a ballistic quantum point contact (QPC), the critical supercurrent $I_c^{\mathrm{QPC}} = \Delta/\hbar$ [22] and the excess normal current $I_{\mathrm{exc}}^{\mathrm{QPC}} = 16\Delta/3h$ [23] are comparable. This normal current can flow at small bias $\Delta\mu/\Delta$ in ballistic channels but not tunnel junctions [23, 24] and can therefore shunt the supercurrent and damp reversible oscillations [25]. If both irreversible and reversible dynamics are significant, then they can both be incorporated into a phenomenological theory in the form of a general equation for non-equilibrium reversible-irreversible coupling (GENERIC) [15, 26].

In the normal system where linear response theory is valid [13, 27], the dissipation potential and currents it generates are

$$\Xi_n = \frac{G}{2T}(\Delta\mu + \alpha_c\Delta T)^2 + \frac{G_T}{2}\left(\frac{\Delta T}{T}\right)^2 = \frac{G}{2T}[(\Delta\mu + \alpha_c\Delta T)^2 + L\Delta T^2] = \Xi_n^a + \Xi_n^d$$
$$\begin{pmatrix} I_N \\ I_S \end{pmatrix} = G\begin{pmatrix} 1 & \alpha_c \\ \alpha_c & L + \alpha_c^2 \end{pmatrix}\begin{pmatrix} \Delta\mu \\ \Delta T \end{pmatrix}. \tag{S17}$$

This model exhibits a linear advective mode of entropy transport $I_S^a = \alpha_c G(\Delta\mu + \alpha_c\Delta T) = \alpha_c I_N$ characterised by the conductance G and Seebeck coefficient α_c, and a linear diffusive mode $I_S^d = G_T\Delta T/T$ characterised by the thermal conductance G_T or equivalently the Lorenz number $L = G_T/TG$.

In the superfluid system, the experimental observation $I_S = s^* I_N$ simplifies the problem of identifying Ξ since it implies the existence of an advective mode Ξ_s^a. Eq. S11 imposes that Ξ_s^a must satisfy $(\partial\Xi_s^a/\partial\Delta T)_{\Delta\mu} = s^*(\partial\Xi_s^a/\partial\Delta\mu)_{\Delta T}$, which has the unique solution $\Xi_s^a(\Delta\mu, \Delta T) = \Xi_s^a(\Delta\mu + s^*\Delta T)$. Based on our observations that I_N is a sigmoidal function of $\Delta\mu$ [28, 29], we make the Ansatz

$$\Xi_s^a = \frac{\sigma I_{\mathrm{exc}}}{T}\log\left[\cosh\left(\frac{\Delta\mu + s^*\Delta T}{\sigma}\right)\right]. \tag{S18}$$

In fact, we find that the simplest form that describes our observations from both the first and second experiment is the sum of the nonlinear advective and linear diffusive modes $\Xi = \Xi_s^a + \Xi_n^d$, which generates the currents

$$I_N = I_{\mathrm{exc}}\tanh\left(\frac{\Delta\mu + \alpha_c\Delta T}{\sigma}\right)$$
$$I_S = \alpha_c I_N + G_T\Delta T/T \tag{S19}$$

where the Seebeck coefficient α_c takes the place of the entropy advectively transported per particle s^*. It is readily verifiable that Ξ_n^a, Ξ_n^d, and Ξ_s^a satisfy the six formal criteria of dissipation potentials [15]. Eq. S19 shows that entropy transport in the superfluid system has the same form as the normal system but the advective mode now responds nonlinearly to the thermodynamic force $\Delta\mu + \alpha_c\Delta T$ and is characterised by the excess current I_{exc} [29], originally defined as the additional current above the normal, Ohmic current in superconducting tunnel junctions [30], and the sharpness of the nonlinearity σ. Ballistic QPCs of superconductors and superfluid ^{3}He exhibit similar current-bias characteristics where the nonlinearity is determined by the highest order multiple Andreev reflection process the QPC can support, i.e., the maximum number of Cooper pairs that can be transported in a single coherent process $\sigma \sim \Delta/n_{\mathrm{pair}}$ [30, 31]. The nonlinearity implies the breakdown of the Wiedemann-Franz law since the advective and diffusive modes are no longer linked. The linear model (Eq. S17) is reproduced in the limit of large σ with $G = I_{\mathrm{exc}}/\sigma$. Our procedure to fit this model to the data is described in Sec. 6. Our fits are also

consistent with the coexistence of both linear and nonlinear advective modes $\Xi = \Xi_s^a + \Xi_n^a + \Xi_n^d$ so we cannot exclude a finite linear advective mode Ξ_n^a but find that the nonlinear advective mode Ξ_s^a adequately describes our observations, yielding reduced chi-square statistics slightly below 1.

3. POTENTIAL ENERGY LANDSCAPE

The total potential energy landscape is a combination of the trap, the channel beams, and the spatially-varying zero point energy of the channel beams' confinement $V(\mathbf{r}) = V_{\text{trap}}(\mathbf{r}) + V_{\text{ch}}(\mathbf{r}) + V_{\text{ZPE}}(y)$. Our trap is a combination of a Gaussian optical dipole trap with wavelength $\lambda = 1064\,\text{nm}$ and a magnetic field oriented along z with harmonic curvature, giving a combined trap potential

$$\begin{aligned} V_{\text{trap}}(\mathbf{r}) &= V_{\text{mag}}(\mathbf{r}) + V_{\text{dip}}(\mathbf{r}) \\ V_{\text{mag}}(\mathbf{r}) &= \frac{1}{2}m(2\pi\nu_{\text{mag},y})^2(x^2 + y^2 - 2z^2) \\ V_{\text{dip}}(\mathbf{r}) &= V_{\text{dip}}^0\left\{1 - \frac{w_{\text{dip},x,0}w_{\text{dip},z,0}}{w_{\text{dip},x}(y)w_{\text{dip},z}(y)}\exp\left[-2\frac{x^2}{w_{\text{dip},x}^2(y)} - 2\frac{z^2}{w_{\text{dip},z}^2(y)}\right]\right\} \end{aligned} \tag{S20}$$

where $w_{\text{dip},x/z}(y)$ follows the usual hyperbolic Gaussian beam width divergence, $\nu_{\text{mag},y} = 28.30(2)\,\text{Hz}$, $w_{\text{dip},x}(0) = w_{\text{dip},z}(0) = 80.7(1)\,\mu\text{m}$, $V_{\text{dip}}^0 = 1.33(1)\,\mu\text{K} \times k_B$. When $\mu, k_BT \ll V_{\text{dip}}^0$, the trap is approximately harmonic with confinement frequencies in all three spatial directions $\nu_{\text{trap},x} = 171(1)\,\text{Hz}$, $\nu_{\text{trap},y} = 28.31(2)\,\text{Hz}$, $\nu_{\text{trap},z} = 164(1)\,\text{Hz}$ and the average trap frequency $\bar{\nu}_{\text{trap}} = 92.7(5)\,\text{Hz}$.

A pair of repulsive TEM_{01}-like beams propagating along x and z, which we call the lightsheet (LS) and wire respectively, intersecting at the centre of the trapped cloud separate it into two reservoirs connected by a channel. The transverse confinement frequencies at their centre are $\nu_z = 9.42(6)\,\text{kHz}$ ($k_BT/h\nu_z = 0.21$) and ν_x ranges from $0.61(1)\,\text{kHz}$ to $12.4(2)\,\text{kHz}$ ($k_BT/h\nu_x \in [0.16, 3.3]$) depending on the power of the beam such that we can explore the crossover from one dimension (1D) to two dimensions (2D) in the channel. An attractive Gaussian beam propagating along z acts as a gate potential in the channel. The combined potential energy landscape of these three beams is

$$\begin{aligned} V_{\text{ch}}(\mathbf{r}) &= V_{\text{LS}}(\mathbf{r}) + V_{\text{wire}}(\mathbf{r}) + V_{\text{gate}}(\mathbf{r}) \\ V_{\text{LS}}(\mathbf{r}) &= f_{\text{LS}}(y)\left\{V_{\text{LS}}^0 + \frac{\pi^3 m(\nu_{\text{LS},z}w_{\text{LS},z})^2}{2}[\text{erfi}(z/w_{\text{LS},z})]^2 f_{\text{LS}}(z)\right\} \\ V_{\text{wire}}(\mathbf{r}) &= f_{\text{wire}}(y)\left\{V_{\text{wire}}^0 + m(\pi\nu_{\text{wire},x}w_{\text{wire}}^{\text{notch}})^2 f_{\text{wire}}(x)\left[1 - f_{\text{wire}}^{\text{notch}}(x)\right]\right\} \\ V_{\text{gate}}(\mathbf{r}) &= f_{\text{gate}}(y)f_{\text{gate}}(x)V_{\text{gate}}^0 \end{aligned} \tag{S21}$$

The envelope function for each beam $f_b(y) \propto P_b e^{-2(y/w_{b,y})^2}$ determine their spatial profile along the respective direction and are proportional to the power of each beam P_b. The finite potential of the LS and wire at the origin $V_{\text{LS/wire}}^0$ arise from optical aberrations that causes their nodal planes to not be perfectly dark, but this is a small correction on the order of 0.1% of the peak potential of each beam. The repulsive LS and wire are generated using blue-detuned 532 nm light while the attractive gate is created with red-detuned 766.7 nm light. The beam waists are $w_{\text{LS},y} = 30.2\,\mu\text{m}$, $w_{\text{LS},z} = 9.5\,\mu\text{m}$, $w_{\text{wire},x} = 78\,\mu\text{m}$, $w_{\text{wire},y} = 6.82\,\mu\text{m}$, $w_{\text{wire}}^{\text{notch}} = 1.5\,\mu\text{m}$, $w_{\text{gate},x} = 30.4\,\mu\text{m}$, $w_{\text{gate},y} = 31.8\,\mu\text{m}$, and the peak confinement frequencies are $\nu_{\text{LS},z} = 9.42(6)\,\text{kHz}$ and $\nu_{\text{wire},x} = 12.4(2)\,\text{kHz}$ at the reference beam powers used for calibration where $f_{\text{LS/wire}}(0) = 1$. The peak gate potential is $V_{\text{gate}}^0 = -2.17(1)\,\mu\text{K} \times k_B$.

The confinement of the channel beams along x and z raise the zero point energy (ZPE) of the atoms, effectively producing a new potential energy along y relative to the ZPE of the trap

$$V_{\text{ZPE}}(y) = \frac{h}{2}\left(\nu_x(y) + \nu_z(y) - (\nu_{\text{trap},x} + \nu_{\text{trap},z})\right) \tag{S22}$$

with the transversal confinement $\nu_x(y) = \sqrt{f_{\text{wire}}(y)\nu^2_{\text{wire},x} + f_{\text{gate}}(y)\nu^2_{\text{gate},x} + \nu^2_{\text{trap},x}}$ and $\nu_z(y) = \sqrt{f_{\text{LS}}(y)\nu^2_{\text{LS},z} + \nu^2_{\text{trap},z}}$ and the confinement frequency of the gate beam given by $\nu_{\text{gate},x} = \sqrt{-V^0_{\text{gate}}/4\pi^2 m w^2_{\text{gate},x}}$. The higher lying modes defined by the transverse confinement that can contribute to transport have energies determined by the quantum numbers of the transverse harmonic oscillator states $n_{x/z} = 0, 1, \ldots$ Their effective potential energy landscape above the trap potential is

$$V_{\text{eff}}(y, n_x, n_z) = V_{\text{ZPE}}(y) + V_{\text{ch}}(0, y, 0) + h\nu_x(y)n_x + h\nu_z(y)n_z \tag{S23}$$

which determines number of occupied modes that contribute to transport

$$n_m = \sum_{n_x,n_z=0}^{\infty} \min_y \frac{1}{1 + \exp\{[V_{\text{eff}}(y, n_x, n_z) - \mu]/k_B T\}}. \tag{S24}$$

The minimum occupation of each mode is used to account for modes that may be occupied in the centre of the channel but are unoccupied elsewhere and therefore non-propagating [27]. The particle conductance of a non-interacting gas through a contact with perfect transparency is $G = 2n_m/h$ [32].

The wall beam, which is only on during preparation and imaging and not during transport, is similar to the wire beam but without the notch cut out

$$V_{\text{wall}}(\mathbf{r}) = f_{\text{wall}}(y)V^0_{\text{wall}}e^{-2(x/w_{\text{wall},x})^2} \tag{S25}$$

where the barrier height V^0_{wall} is larger than μ and $k_B T$ to completely block transport. Its width is large enough to completely suppress tunnelling [33].

The complete potential energy landscape $V(\mathbf{r})$ was used to produce Fig. 1a via the local density approximation for the density $n(\mathbf{r}) = n[\mu - V(\mathbf{r}), T]$ [34, 35] that determines the local Fermi temperature $k_B T_F(\mathbf{r}) = \hbar^2[3\pi^2 n(\mathbf{r})]^{2/3}/2m$. For weak wire beam powers, the transverse confinement frequency at the centre of the channel $\nu_x = \nu_x(y = 0)$ is small and the barriers at the edges of the cloud disappear. In this case, the equilibrium degeneracy varies slowly along x from deeply degenerate and superfluid in the centre of the channel to weakly degenerate and normal at the edges of the channel to vacuum far from the centre. Fig. S1a shows how the degeneracy varies with x (in units of the thermal de Broglie wavelength $\lambda_T = h/\sqrt{2\pi m k_B T} = 2.4\,\mu\text{m}$) around $\mathbf{r} = 0$ for various powers of the wire beam. The horizontal dashed line is the critical degeneracy at the superfluid transition $(\mu/k_B T)_c = 2.49$ [34]. This shows that in the 1D channel, the only occupied modes are tightly confined and expected to be superfluid at equilibrium, while in the 2D channel, weakly confined and normal transport modes appear at the edges. While the location of the 1D-2D transition is not well-defined, the channel is significantly wider at the occupation threshold $\mu - V(\mathbf{r}) = 0$ for $\nu_x \lesssim 7\,\text{kHz}$. This is approximately the same ν_x as the centre location of the feature resembling an avoided crossing in Fig. 4d, which is determined by the dimensional crossover. The 2D contacts to the 1D channel are the most degenerate regions in the system in equilibrium, see Fig. 1a. We estimate the superfluid gap here using the local density approximation applied to a calculation of the gap in a homogeneous system $\Delta(\mu_c, T)$ [36] where $\mu_c = \max_{\mathbf{r}}[\mu - V(\mathbf{r})]$.

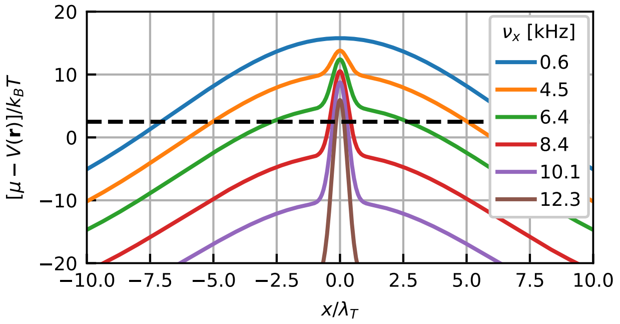

FIG. S1. **1D-2D crossover of the channel.** The equilibrium local degeneracy at $y = z = 0$ vs. x in units of the thermal de Broglie wavelength $\lambda_T = h/\sqrt{2\pi m k_B T} = 2.4\,\mu\mathrm{m}$ for various powers of the wire beam shows that for high powers (large ν_x), the channel is tightly confining and there are few occupied modes (regions with degeneracy > 0) while for low powers (small ν_x), the confinement is very weak and there are many occupied modes. Since, in the 2D limit, the potential varies over a much larger length scale than λ_T, we can apply a local density approximation picture and identify regions at large x (the edges of the channel) which are occupied and can contribute to transport but below the critical degeneracy to be superfluid (horizontal dashed line).

4. PREPARATION OF THE INITIAL STATE

As part of the experimental sequence, we ramp up the channel beams to separate the two reservoirs then performed forced optical evaporation before allowing transport by opening the wall. Using a magnetic field gradient along y, we can shift the centre of the magnetic trap with respect to the channel beams before separation to prepare $\Delta N(0)$ before shifting the centre back to coincide with the channel beams for transport, which ensures that $\Delta N = 0$ at equilibrium $\Delta\mu = 0$. By shifting the centre after separation to a different position during evaporation, we can compress one reservoir and decompress the other, thereby changing their evaporation efficiencies and inducing a controllable $\Delta S(0)$ as well. The average efficiency changes only slightly with the imbalances such that the net entropy per particle S/Nk_B varies by < 9% over the large range of $\Delta N(0)$ and $\Delta S(0)$ we prepare. This method achieves much lower entropy–therewith enabling the study of entropy transport in the superfluid phase–than a previously used method [13, 27, 37] where evaporation was performed in the same trap as separation, but additional entropy was injected into one reservoir by focusing the gate beam into the reservoir and modulating the power.

Between the end of transport and the start of imaging, we ramp down the channel beams while keeping the wall on. Typical images of the cloud at each of these stages are shown in Fig. S2. For the experiments at all ν_x that contain only the advective modes (Fig. 2), we prepare a density imbalance $\Delta N(0)$ and the corresponding entropy imbalance $\Delta S(0)$ such that the system relaxes to equilibrium in the 1D channel $\nu_x = 12.4(2)\,\mathrm{kHz}$ at long times and not the NESS. For the experiments with both the advective and diffusive modes (Fig. 3), we fix $\Delta N(0) = 0$ and prepare as large $\Delta S(0)$ as possible without losing atoms.

To measure the spin conductance G_σ, we employ a similar method as in [38] though instead of quenching the gradient along y, we modulate it at the average trap frequency for the two spin states for half the period of the trap frequency difference to induce a larger initial spin imbalance. We use the recently computed equation of state of the finite-temperature polarised unitary fermi gas [39] to compute a spin susceptibility $\chi \approx 0.32\kappa$ [29] in agreement with measurements on a harmonically-trapped unitary fermi gas at low temperatures [40].

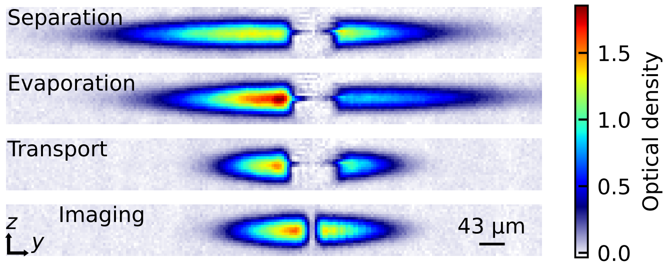

FIG. S2. **Absorption images of the system at various stages of the experiment**: just after separation into two reservoirs, during evaporation, during transport without the gate beam, and during imaging. Each image is an average over 10 shots of spin $|\uparrow\rangle$ and the magnified pixel size is 4.3 µm.

5. RESERVOIR THERMODYNAMICS

A. Effect of the potential energy landscape

The potential energy landscape $V(\mathbf{r})$ described in Sec. 3 determines the equilibrium thermodynamic properties of the reservoirs. In the local density approximation [35], the grand canonical free energy of the full system (both spins in both reservoirs) is given in terms of the universal pressure equation of state of the unitary fermi gas [34]

$$\Omega(\mu, T) = -\frac{k_B T}{\lambda_T^3} \int \mathrm{d}^3 r\, f_P\left[\frac{\mu - V(\mathbf{r})}{k_B T}\right]. \tag{S26}$$

While Ω takes a simple form for a harmonic trap, the trap is highly anharmonic during transport due to the channel beams (especially the lightsheet) and is slightly anharmonic during imaging due to the wall and gaussian dipole trap. It is therefore a non-universal function of μ, T, and the powers of the various beam $\Omega(\mu, T, P_{\mathrm{dip}}, P_{\mathrm{LS}}, P_{\mathrm{wire}}, P_{\mathrm{gate}}, P_{\mathrm{wall}})$. The magnetic trap frequency $\nu_{y,\mathrm{mag}}$ is fixed at unitarity by the geometry of the Feshbach coils. In a given trap defined by these powers, the extensive quantities and response functions are given by derivatives of Ω

$$\begin{aligned} N &= -\left(\frac{\partial \Omega}{\partial \mu}\right)_T & \kappa &= -\frac{\partial^2 \Omega}{\partial \mu^2} \\ S &= -\left(\frac{\partial \Omega}{\partial T}\right)_\mu & \alpha_r &= -\frac{1}{\kappa}\frac{\partial^2 \Omega}{\partial \mu \partial T} \\ U &= \Omega + \mu N + TS & \ell_r &= -\frac{1}{\kappa}\frac{\partial^2 \Omega}{\partial T^2} - \alpha_r^2 \end{aligned} \tag{S27}$$

As explained in Sec. 3 and shown in Fig. S2, the potential energy landscape $V(\mathbf{r})$ differs between transport, where we wish to know the response coefficients, and imaging, where we measure N_i, S_i, so the EoS of the gas $\Omega(\mu, T)$ is different in these two conditions. However, the two are connected because N_i, S_i are the same in both the transport and imaging conditions as a consequence of the adiabaticity of the channel beam ramps. This fact can be used to determine the chemical potential and temperature in each reservoir during transport $\mu_i^{\mathrm{tr}}, T_i^{\mathrm{tr}}$ from their values extracted in the imaging condition $\mu_i^{\mathrm{im}}, T_i^{\mathrm{im}}$ using the EoS of each reservoir in the two configurations $\Omega^{\mathrm{tr}}(\mu_i^{\mathrm{tr}}, T_i^{\mathrm{tr}})/2$ and $\Omega^{\mathrm{im}}(\mu_i^{\mathrm{im}}, T_i^{\mathrm{im}})/2$ that can be computed from the known $V^{\mathrm{tr}}(\mathbf{r})$ and $V^{\mathrm{im}}(\mathbf{r})$. The response coefficients can then be computed from $\mu^{\mathrm{tr}} = (\mu_L^{\mathrm{tr}} + \mu_R^{\mathrm{tr}})/2$, $T^{\mathrm{tr}} = (T_L^{\mathrm{tr}} + T_R^{\mathrm{tr}})/2$, and $\Omega^{\mathrm{tr}}(\mu^{\mathrm{tr}}, T^{\mathrm{tr}})$.

The lightsheet has the strongest influence on the reservoirs' EoS since it is large in comparison to the cloud as seen in Fig. S2. Its primary effect is to increases the chemical potential by pushing the cloud to the edges of the trap where the potential energy is larger. We observe this directly by imaging the cloud in the transport configuration for various lightsheet beam powers and see that, while the atom number in the system remains constant, atoms are pushed away from the central region. The gate has a weaker but still significant effect at the strong lightsheets used here: it draws about 7% of the total number of atoms into the channel independently of ν_x. The local density approximation with the 3D EoS f_P overestimates the fraction of atoms drawn into the channel since the system is in fact quasi-2D or quasi-1D in most of the channel where the density is lower at the same μ and T [41, 42], though this has a small effect on the overall thermodynamics as the 2D and 1D regions are small. Using the procedure outlined above, we estimate the ratio of several thermodynamic properties during transport to their values during imaging. We estimate that T^{tr} is within 1% of T^{im} while μ^{tr} is 24% larger than μ^{im}. Furthermore, κ^{tr} is within 1% of κ^{im} but α_r^{tr} and ℓ_r^{tr} are respectively 3.4 and 2.6 times larger than their values during imaging. In short, T and κ are essentially the same during transport as during imaging, with the other parameters, especially the response functions, are much more sensitive to the trap potential. This is consistent with our observation that the conductance of the weakly-interacting gas is quantised in units of $1/h$ which relies on an accurate value of κ without incorporating these corrections [32]. Motivated by this finding and the fact that the potential energy landscape we estimate is idealised and not exact (there are clear asymmetries in the lightsheet in Fig. S2), we fix T and κ to the measured values but fit α_r and ℓ_r along with the other parameters of the model (Sec. 6).

B. Thermometry from absorption images

It is not straightforward to apply standard thermometry techniques [43] to absorption images of the reservoirs in the transport configuration including all the channel beams (see Fig. S2) since it is difficult to precisely characterise the complex potential energy landscape that determines the reservoirs' EoS. We therefore ramp the channel beams down between the end of transport and the beginning of imaging such that the potential energy landscape is well-known and harmonic. This ramp is performed adiabatically such that the atom number and entropy in each reservoir remains constant even though their energies change due to the work done by the time-varying beams. This was confirmed by ensuring that N_i and S_i/N_ik_B are the same for slower ramps.

At the end of each shot, we obtain the column density $n_{i\sigma}^{\mathrm{col}}(y,z)$ of both reservoirs $i = L, R$ and both spin states $\sigma = \downarrow, \uparrow = |1\rangle, |3\rangle$ from two absorption images 225 µs apart taken *in situ* along the x axis with a calibrated imaging system. The atom number in each spin state and reservoir is determined by integrating the column density over the half-plane of each reservoir

$$\begin{aligned} N_{L\sigma} &= \int_{-\infty}^{0} \mathrm{d}y \int_{-\infty}^{\infty} \mathrm{d}z\, n_{L\sigma}^{\mathrm{col}}(y,z) \\ N_{R\sigma} &= \int_{0}^{\infty} \mathrm{d}y \int_{-\infty}^{\infty} \mathrm{d}z\, n_{R\sigma}^{\mathrm{col}}(y,z). \end{aligned} \tag{S28}$$

The centre $y = 0$ is fixed to the centre of a gaussian fit to the density profile in the wall region. We fit the degeneracy $q_{i\sigma} = \mu_{i\sigma}/k_BT_{i\sigma}$ and temperature $T_{i\sigma}$ of both reservoirs for each spin state using the EoS of the harmonically-trapped gas

$$n_{i\sigma}^{\mathrm{col}}(y,z) = \lambda_{Ti\sigma}^{-3} \int_{-\infty}^{\infty} \mathrm{d}x\, f_n\left[q_{i\sigma} - \left(\frac{x}{R_{xi\sigma}}\right)^2 - \left(\frac{y}{R_{yi\sigma}}\right)^2 - \left(\frac{z - z_0}{R_{zi\sigma}}\right)^2 \right] \tag{S29}$$

where $\lambda_{Ti\sigma} = h/\sqrt{2\pi m k_B T_{i\sigma}}$ is the thermal de Broglie wavelength, f_n is the universal density EoS of a single spin in a balanced unitary Fermi gas [34], and $R_{ji\sigma}^2 = 2k_BT_{i\sigma}/m(2\pi\nu_{\mathrm{trap},j})^2$ is

the Gaussian thermal length given by the known trap frequencies and the fitted temperature. In practice, we do not fix R_z to R_y via the trap frequencies and the common temperature, but rely only on the calibrated and almost harmonic magnetic trap frequency along y. We exclude the region $\approx 60\,\mu\text{m}$ wide containing the wall from the fit, fix both reservoirs and spin states to have a common centre $y = 0$ fixed to the fitted centre of the wall, and fit z_0 with a common value for the two reservoirs. We also fit a small rotation angle between the camera's CCD grid and the harmonic trap principle directions, which we find to be $0.45(5)°$. We estimate that, at the same μ and T, the gas with the wall of finite width has S/Nk_B that is $< 2\%$ larger than the half-harmonic approximation wherein the wall is assumed to have zero width. While the atom numbers match for the two spin states, we see that the fitted q for the second spin state to be imaged (regardless of which spin is imaged first) is systematically larger, likely due to off-resonant photon scattering from the first pulse and energy transfer from collisions with the the first spin. We therefore use the thermodynamics of the first spin state. This thermometry method based on directly fitting $q_{i\sigma}$ and $T_{i\sigma}$ gives similar results as a previously-used method using the second spatial moment of the reservoirs' column density distributions to extract thermodynamic properties [13].

C. Calibration of absorption images

The absorption images of the column density are determined from raw image containing atoms $A(y,z)$ and a "bright" reference image $B(y,z)$

$$\frac{\sigma_0}{\alpha} n_{\text{col}}(y,z) = \text{OD}(y,z) = \log \frac{B(y,z)}{A(y,z)} + \frac{B(y,z) - A(y,z)}{\chi t} \tag{S30}$$

where σ_0 is the resonant absorption cross section, α is a correction of the absorption cross section, χ is the imaging transition's saturation intensity I_{sat} in units of the camera count rate, and t is the pulse duration [44, 45]. For each shot, we use the optimal reference image B [46] to suppress photon shot noise and technical noise, though the latter contribution is negligible. The duration of the imaging pulses was $t = 2\,\mu\text{s}$ to avoid any Doppler shifts, which we verified by ensuring that the resonance frequency does not depend on the pulse power. We calibrated χ by ensuring the measured atom number does not depend on the imaging intensity I [47]; we ultimately used $I/I_{\text{sat}} \approx 0.75$.

The correction α is typically calibrated by imaging a very degenerate cloud and fixing the amplitude of the OD using the equation of state [44, 48], however the clouds that we prepare are not sufficiently degenerate to robustly fit q and α independently. We therefore calibrated α by measuring the onset of Bose-Einstein condensation after an adiabatic sweep of the magnetic field to the BEC side of the Feshbach resonance [43] as shown in Fig. S3. We prepared harmonically trapped gases (no channel beams) at varying levels of degeneracy by varying the depth of the optical dipole trap at the end of evaporation and then either imaged *in situ* to perform the thermometry procedure described in 5 B or adiabatically swept the magnetic field over 50 ms to the BEC regime and imaged the cloud after a 6 ms time of flight. The magnetic field at the end of the sweep is 611.1 G where the scattering length between fermions of opposite spin is $a = 904a_0$ [49], $1/k_F a = 7.4$, and the scattering length between the Feshbach molecules is $0.6a$ [43]. For non-degenerate clouds (a,b), the density distribution after the expansion is gaussian and therefore not condensed, however for degenerate clouds (c,d), the density is bimodal and therefore the entropy per fermion is below the critical value $(S/Nk_B)_{c0} = 1.801$ for a non-interacting Bose gas in a harmonic trap [11]. The condensate fraction N_0/N measured on the BEC side, which is independent of α and χ, vs. the entropy per particle measured at unitarity (e) shows the critical point as a kink at the transition between where the condensate fraction is flat to where it monotonically increases as S/Nk_B decreases. The cloud is distorted due to the sweep of the magnetic trap frequency, so a finite condensate fraction is always fitted, and the critical entropy

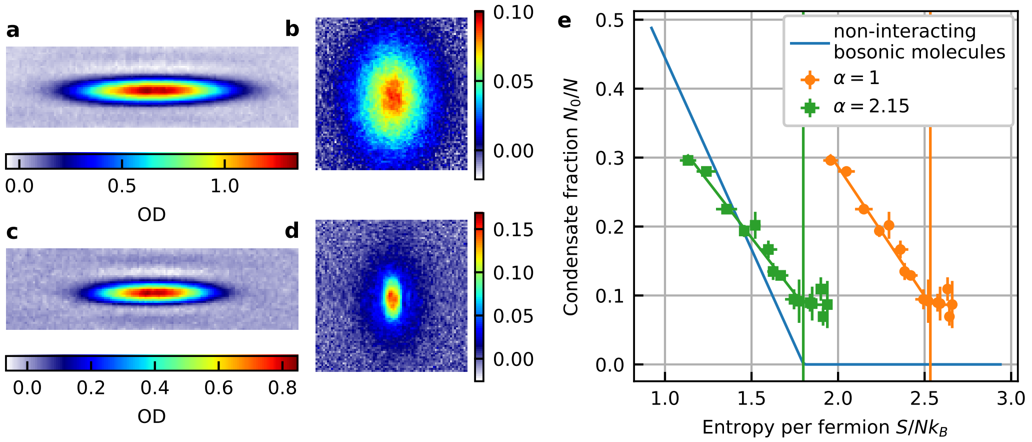

FIG. S3. **Calibration of the absorption images via Bose-Einstein condensation of molecules.** **a**, Average of 5 absorption images of a single spin acquired *in situ* in the harmonic trap at unitarity with a relatively deep dipole trap at the end of the forced optical evaporation to prepare a non-degenerate cloud. **b**, The same system prepared in **a** after an adiabatic sweep of the magnetic field to the BEC side of the Feshbach resonance followed by a short time of flight. The density distribution is well-fitted by a gaussian and is therefore above the critical point for Bose-Einstein condensation. **c**, Same as **a** but with a lower dipole trap depth at the end of evaporation to prepare a more degenerate cloud. **d**, Same as **c** after the magnetic field sweep and time of flight, displaying the bimodal density distribution that indicates Bose-Einstein condensation of Feshbach molecules. **e**, The condensate fraction measured on the BEC side vs. the entropy per fermion measured at unitarity. The solid line shows the expected behaviour for a non-interacting Bose gas, the orange circles show the measurement with $\alpha = 1$, and the green squares show the measurement when correcting the absorption cross section with $\alpha = 2.15$ to match the measured and theoretical critical points.

per fermion is set by the kink rather rather then the extrapolated x-intercept. Assuming the theoretical absorption cross section ($\alpha = 1$), we find the measured critical entropy per particle $(S/Nk_B)_c$ to be larger than $(S/Nk_B)_{c0}$, indicating that the true entropy per particle is lower than our uncorrected thermometry indicates. We scale the cross section α to 2.15 such that $(S/Nk_B)_c = (S/Nk_B)_{c0}$ at the onset of condensation. We have verified that slower sweeps, sweeping further into the BEC regime with smaller scattering length, and longer time of flight do not change the measured condensate fraction. The reduced slope of N_0/N vs. S/Nk_B relative to the theoretical prediction may be due to residual weak repulsive interactions [11] or heating and three-body losses during this sweep. Both these effects also shift the transition to a lower $(S/Nk_B)_c$, so the true S/Nk_B is likely slightly smaller than this calibration procedure indicates. The direct fit of α with the EoS is consistent with 2.15, but the systematic uncertainty is larger due to the low degeneracy of the clouds. We have also cross-checked this value of α calibrated at unitarity by measuring conductance quantisation to $1/h$ in the non-interacting gas, which relies on accurate thermometry to compute the compressibility [32].

The calibrated value $\alpha = 2.15$ is similar to previously reported values [44, 48] though the origin of this large correction factor is not completely clear. It is important to note that miscalibration of the imaging system's magnification and in χ is absorbed into α, though we expect these corrections to be small: the 1% (4%) relative uncertainty in magnification (χ) leads to 3% (3%) relative uncertainty in S/Nk_B. χ/α can be independently calibrated with the camera's magnified pixel size, quantum efficiency, gain, and the transmission of the imaging system [44, 45] though we find that this value is 50% larger than the above calibration procedure predicts. It is therefore

likely that there are still some non-ideal effects that are not explicitly accounted for in Eq. S30 but are approximately absorbed into χ and α. Indeed, as in [44], we measure higher atom numbers at lower intensities, which would reduce the value of α required to match $(S/Nk_B)_c = (S/Nk_B)_{c0}$, and we observe the speckle pattern of the beam being partially visible in the OD at both low and high intensities. We can reasonably rule out density effects on the absorption cross section, e.g. from multiple scattering of photons [50], by verifying that the measured atom number is independent of the time of flight after release from the optical trap which significantly varies the density of the gas at fixed atom number. Imperfections of the beam's polarisation can increase α, though this effect should be small as we measured with a polarisation analyser that the polarisation is within 1° of the expected horizontal polarisation before and after the vacuum chamber. Even though we use short pulses, the Doppler shift can still be significant: Not only do the atoms acquire an average Doppler shift due to the absorbed photon recoil, they also develop an increasingly broad distribution due to the stochasticity in the direction of the re-emitted photons [51]. We estimate that this can increase α by 10-20%. For the short pulses we use, the atoms are not in the steady internal state that is assumed by Eq. S30; we find by solving the Bloch equations for the 6 ground states and the 18 excited states in the D_1 and D_2 lines, we find that the number of scattered photons and therefore σ is reduced by about 5% relative to the steady state solution. The laser spectrum, both its lorentzian line and broad background typical of diode lasers, can also increase α by 5-10%. Optical effects such as aberrations and refraction by the second spin state are also present but their effect on absorption imaging is rarely considered. A complete explanation of the deviation of α and χ from their expected values is beyond the scope of this study and we rely on the condensate fraction as an absolute calibration of S/Nk_B. Moreover, none of our conclusions change qualitatively when not implementing this calibration procedure, only the value α_c becomes a factor of ≈ 2 larger and I_{exc} is 2.15 times smaller.

6. FITTING PROCEDURE

We fit the phenomenological model to each data set–the set of different transport times at fixed ν_x–independently for both the first and second experiment. We do this by solving the initial value problem for $\Delta N(t)$ and $\Delta S(t)$

$$\begin{aligned}
\frac{\mathrm{d}\Delta N(t)}{\mathrm{d}t} &= -2I_N[\Delta\mu(t), \Delta T(t)] = -2\left\{I_{\text{exc}} \tanh\left[\frac{\Delta\mu(t) + \alpha_c \Delta T(t)}{\sigma}\right] + G[\Delta\mu(t) + \alpha_c \Delta T(t)]\right\} \\
\frac{\mathrm{d}\Delta S(t)}{\mathrm{d}t} &= -2I_S[\Delta\mu(t), \Delta T(t)] = -2\{\alpha_c I_N[\Delta\mu(t), \Delta T(t)] + G_T \Delta T(t)/T\} \\
\begin{pmatrix}\Delta\mu(t) \\ \Delta T(t)\end{pmatrix} &= \frac{2}{\kappa\ell_r}\begin{pmatrix}\ell_r + \alpha_r^2 & -\alpha_r \\ -\alpha_r & 1\end{pmatrix}\begin{pmatrix}\Delta N(t) \\ \Delta S(t)\end{pmatrix}
\end{aligned} \tag{S31}$$

given the parameters G, α_c, I_{exc}, σ, G_T along with the reservoir response functions κ, α_r, ℓ_r and average temperature T during transport and the initial values $\Delta N(0)$, $\Delta S(0)$. From these solutions, we also compute the total entropy as a function of time

$$S(t) = S_{\text{eq}} - \frac{\Delta N^2(t)}{2T\kappa} - \frac{[\Delta S(t) - \alpha_r \Delta N(t)]^2}{2T\ell_r\kappa} \tag{S32}$$

where S_{eq} is the equilibrium total entropy. From the data–the set of times t_i, relative particle imbalances $\Delta N_i/N_i$, relative entropy imbalances $\Delta S_i/N_i k_B$, and relative total entropies

S_i/N_ik_B–we perform a least-squares fit by minimising the reduced chi-squared statistic

$$\chi^2 = \frac{1}{\nu}\sum_i \left(\frac{\Delta N_i/N_i - \Delta N(t_i)/N}{\sigma_{\Delta N/N}}\right)^2 + \left(\frac{\Delta S_i/N_ik_B - \Delta S(t_i)/Nk_B}{\sigma_{\Delta S/Nk_B}}\right)^2 + \left(\frac{S_i/N_ik_B - S(t_i)/Nk_B}{\sigma_{S/Nk_B}}\right)^2. \quad \text{(S33)}$$

In other words, we simultaneously fit the particle imbalance, entropy imbalance, and total entropy. ν is the number of degrees of freedom: the number of data points minus number of fit parameters. For each quantity $x = \Delta N/N, \Delta S/Nk_B, S/Nk_B$, we use the same uncertainty for each data point defined as the average over each point's statistical uncertainty computed from 3-5 shots $\sigma_x = \sum_i \sigma_{x,i}/n_{\text{points}}$ in order to improve the stability of the fits. We use the relative quantities normalised by the atom number (e.g. $\Delta N/N$ instead of ΔN) to minimise the effect of $\sim 10\,\%$ shot-to-shot fluctuations of the total atom number.

For both experiments, we set $G = 0$ as allowing G to be a fit parameter underconstrains the fit (G is strongly correlated to I_{exc}/σ) and enforcing $G = 2n_m/h$ increases χ^2 by $\sim 20\%$. We also fix $\kappa = \kappa^{\text{tr}}(\mu^{\text{tr}}, T^{\text{tr}})$ to the value computed from the equation of state in the transport trap (see Sec. 5 A). For the first experiment, we fit I_{exc}, σ, α_c, $\Delta S(0)$, and S_{eq}, and fix $G_T = 0$ (the dynamics are dominated by the advective mode), $\alpha_r = \alpha_r^{\text{tr}}(\mu^{\text{tr}}, T^{\text{tr}})$, and $\ell_r = \ell_r^{\text{tr}}(\mu^{\text{tr}}, T^{\text{tr}})$. ΔS is offset by the value at long times where equilibrium is reached. This offset is a systematic bias in the thermometry due to drifts in the alignment. For the second experiment, we fit σ, $\Delta S(0)$, S_{eq}, G_T, α_r, and ℓ_r. As equilibrium is not reached at the longest time for $\nu_x > 6$ kHz, we also fit an offset in ΔS to account for misalignment. Furthermore, we fix $\alpha_c = 1.18k_B$ to the average fit result from the first experiment since only $\alpha_c - \alpha_r$ is important in the second experiment and we fix I_{exc} to the initial current $I_N(0)$ determined by a linear fit to all points in the initial response with $\Delta N/N \le 0.07$. Without fixing I_{exc} in this way, it is strongly correlated to σ and G_T and yields similar values of χ^2. Fixing α_r and ℓ_r to their theoretical values prevents the fit from reproducing the large ΔN that is induced by the initial entropy imbalance $\Delta S(0)$ in the second experiment.

With this method, the resulting $\chi^2 \approx 1$ for all data sets. The fitted values of the reservoir response coefficients from the second experiment are shown in Fig. S4. While α_r is relatively near the expected value for the anharmonic trap, ℓ_r is significantly smaller. There can be many reasons for these systematic biases, for example miscalibration of the absorption cross section and magnification, the contacts defined by the gate beam and wire forming smaller reservoirs contacted to the larger ones which develop their own effective thermodynamic response functions, or generally deviations of the true equation of state from the computed one. Despite this deviation from our expectations, this result does not change the overall form of the phenomenological model nor the central result of the large advective entropy current. We find that fixing α_r and ℓ_r in the first experiment yield good fits while they must be allowed to vary in the second experiment to fit the data. Allowing them to vary in the first experiment does not change the extracted parameters I_{exc} and α_c. The average fitted nonlinearity from all the data sets is $\sigma = 7.2(5)\,\text{nK} \times k_B = 4.4(5) \times 10^{-3}\Delta$.

We can derive an expression for the instantaneous slope of the path through state space $\Pi = \mathrm{d}\Delta S/\mathrm{d}\Delta N$ by combining the expression for I_S in Eq. S19 and the reservoir responses in Eq. S13

$$\Pi = \frac{\mathrm{d}\Delta S}{\mathrm{d}\Delta N} = \frac{\mathrm{d}\Delta S/\mathrm{d}t}{\mathrm{d}\Delta N/\mathrm{d}t} = \alpha_c + \frac{4G_T(\alpha_r\Delta N - \Delta S)}{T\kappa\ell_r\,\mathrm{d}\Delta N/\mathrm{d}t} \quad \text{(S34)}$$

In the limit where either the advective or diffusive mode dominates, the path is linear $\Delta S(t) \approx \Pi[\Delta N(t) - \Delta N_0]$ [Figs. 1d and 3d]. When combined with the previous expression, this gives

$$\Pi \approx \frac{4G_T\alpha_r\Delta N + T\kappa\ell_r\alpha_c\,\mathrm{d}\Delta N/\mathrm{d}t}{4G_T(\Delta N - \Delta N_0) + T\kappa\ell_r\,\mathrm{d}\Delta N/\mathrm{d}t} \quad \text{(S35)}$$

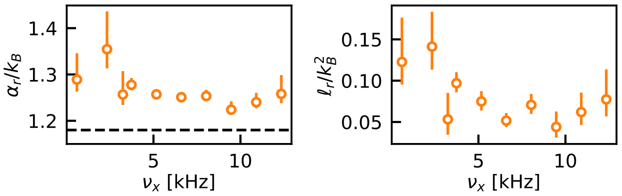

FIG. S4. Fitted reservoir response coefficients for each configuration of the second experimentThe dashed horizontal line indicates the fixed α_c value in these fits.

If the transport is fast as is the case for the superfluid-induced advective mode, we observe that Π is independent of ΔN, implying that the $\mathrm{d}\Delta N/\mathrm{d}t$ terms dominate and the slope simplifies to

$$\Pi_a \approx \alpha_c. \tag{S36}$$

We observe in the second experiment that after the advective mode has quickly relaxed and the diffusive mode is dominant, then the time evolution is exponential $\mathrm{d}\Delta N/\mathrm{d}t \approx -\Delta N/\tau_d$, indicating that the response becomes linear in this regime $I_{\mathrm{exc}} \tanh[(\Delta\mu + \alpha_c \Delta T)/\sigma] \approx I_{\mathrm{exc}}(\Delta\mu + \alpha_c\Delta T)/\sigma$. Linearising tanh is equivalent to applying the linear model $\Xi = \Xi_n^a + \Xi_n^d$ with effective conductance $\tilde{G} = I_{\mathrm{exc}}/\sigma + G$ and Lorenz number $L = G_T/T\tilde{G}$

$$\frac{\mathrm{d}}{\mathrm{d}t}\begin{pmatrix}\Delta N(t)\\ \Delta S(t)\end{pmatrix} \approx -2\tilde{G}\begin{pmatrix}1 & \alpha_c\\ \alpha_c & L+\alpha_c^2\end{pmatrix}\begin{pmatrix}\Delta\mu(t)\\ \Delta T(t)\end{pmatrix} = -\frac{4\tilde{G}}{\kappa\ell_r}\begin{pmatrix}1 & \alpha_c\\ \alpha_c & L+\alpha_c^2\end{pmatrix}\begin{pmatrix}\ell_r+\alpha_r^2 & -\alpha_r\\ -\alpha_r & 1\end{pmatrix}\begin{pmatrix}\Delta N(t)\\ \Delta S(t)\end{pmatrix} = -\mathbf{\Lambda}\begin{pmatrix}\Delta N(t)\\ \Delta S(t)\end{pmatrix}. \tag{S37}$$

In this limit, the path the system traces though state space is a line passing through equilibrium and can be written

$$\begin{pmatrix}\Delta N(t)\\ \Delta S(t)\end{pmatrix} = \Delta N(0)e^{-t/\tau_d}\begin{pmatrix}1\\ \Pi_d\end{pmatrix} \tag{S38}$$

so the exponential timescale τ_d and the slope of the path Π_d are then given by the smaller eigenvalue and corresponding eigenvector of the matrix $\mathbf{\Lambda}$

$$\begin{aligned}
\tau_d^{-1} &= \frac{2\tilde{G}}{\kappa\ell_r}\left\{L + \ell_r + (\alpha_c-\alpha_r)^2 - \sqrt{[L+\ell_r+(\alpha_c-\alpha_r)^2]^2 - 4L\ell_r}\right\}\\
&= \frac{4\tilde{G}}{\kappa\ell_r}[\ell_r + (\alpha_r-\alpha_c)(\alpha_r-\Pi_d)]\\
\Pi_d &= \frac{L-\ell_r+\alpha_c^2-\alpha_r^2-\sqrt{[L+\ell_r+(\alpha_c-\alpha_r)^2]^2-4L\ell_r}}{2(\alpha_c-\alpha_r)}\\
&= \frac{4\tilde{G}[\ell_r+\alpha_r(\alpha_r-\alpha_c)]+\kappa\ell_r\tau_d^{-1}}{4\tilde{G}(\alpha_r-\alpha_c)}.
\end{aligned} \tag{S39}$$

In the non-equilibrium steady state, $\tau_d^{-1} \to 0$ which implies that $[L+\ell_r+(\alpha_c-\alpha_r)^2]^2 \gg 4L\ell_r$, i.e., $L \to 0$. Taylor expanding τ_d^{-1} and Π_d to first order in L yields

$$\begin{aligned}
\tau_d^{-1} &\approx \frac{4\tilde{G}L}{\kappa[\ell_r+(\alpha_c-\alpha_r)^2]}\\
\Pi_d &\approx \alpha_r + \frac{\ell_r}{\alpha_r-\alpha_c}\left[1-\frac{L}{\ell_r+(\alpha_r-\alpha_c)^2}\right].
\end{aligned} \tag{S40}$$

As first observed in [13], we see that L decreases with increasing ν_x from Fig. 3 where the diffusive timescale τ_d becomes longer and from Fig. 4d where Π_d increases.

---

[1] Khalatnikov, I. M. *An introduction to the theory of superfluidity*. Advanced book classics (Advanced Book Program, Perseus Pub, Cambridge, Mass, 2000).

[2] Svistunov, B. V., Babaev, E. S. & Prokof'ev, N. V. *Superfluid States of Matter* (CRC Press, 2015), 0 edn. URL https://www.taylorfrancis.com/books/9781439802762.

[3] Golubov, A. A., Kupriyanov, M. Y. & Il'ichev, E. The current-phase relation in Josephson junctions. *Reviews of Modern Physics* **76**, 411–469 (2004). URL https://link.aps.org/doi/10.1103/RevModPhys.76.411.

[4] Sols, F. & Ferrer, J. Crossover from the Josephson effect to bulk superconducting flow. *Physical Review B* **49**, 15913–15919 (1994). URL https://link.aps.org/doi/10.1103/PhysRevB.49.15913.

[5] Tinkham, M. *Introduction to superconductivity*. International series in pure and applied physics (McGraw Hill, New York, 1996), 2nd ed edn.

[6] Hoskinson, E., Sato, Y., Hahn, I. & Packard, R. E. Transition from phase slips to the Josephson effect in a superfluid ^{4}He weak link. *Nature Physics* **2**, 23–26 (2006). URL https://www.nature.com/articles/nphys190.

[7] Varoquaux, E. Anderson's considerations on the flow of superfluid helium: Some offshoots. *Reviews of Modern Physics* **87**, 803–854 (2015). URL https://link.aps.org/doi/10.1103/RevModPhys.87.803.

[8] Yan, Z. *et al.* Thermography of the superfluid transition in a strongly interacting Fermi gas. *Science* **383**, 629–633 (2024). URL https://www.science.org/doi/10.1126/science.adg3430.

[9] Wang, X., Li, X., Arakelyan, I. & Thomas, J. E. Hydrodynamic Relaxation in a Strongly Interacting Fermi Gas. *Physical Review Letters* **128**, 090402 (2022). URL https://link.aps.org/doi/10.1103/PhysRevLett.128.090402.

[10] Li, X. *et al.* Second sound attenuation near quantum criticality. *Science* **375**, 528–533 (2022). URL https://www.science.org/doi/10.1126/science.abi4480.

[11] Pitaevskii, L. P. & Stringari, S. *Bose-Einstein condensation* (Oxford University Press, Oxford, United Kingdom, 2003), first edition edn.

[12] Donnelly, R. J. The two-fluid theory and second sound in liquid helium. *Physics Today* **62**, 34–39 (2009). URL https://pubs.aip.org/physicstoday/article/62/10/34/1017242/The-two-fluid-theory-and-second-sound-in-liquid.

[13] Husmann, D. *et al.* Breakdown of the Wiedemann–Franz law in a unitary Fermi gas. *Proceedings of the National Academy of Sciences* 201803336 (2018). URL http://www.pnas.org/content/early/2018/08/07/1803336115.

[14] Balian, R. *From Microphysics to Macrophysics: Methods and Applications of Statistical Physics*, vol. 2 (Springer, 2007).

[15] Pavelka, M., Klika, V. & Grmela, M. *Multiscale thermo-dynamics: introduction to GENERIC* (De Gruyter, Berlin ; Boston, 2018).

[16] Landau, L. & Lifshitz, E. *Statistical Physics Part I*, vol. 5 of *Course of Theoretical Physics* (Pergamon Press, 1969), second revised and enlarged edition edn.

[17] Grenier, C., Kollath, C. & Georges, A. Thermoelectric transport and Peltier cooling of cold atomic gases. *Comptes Rendus Physique* **17**, 1161–1174 (2016). URL http://www.sciencedirect.com/science/article/pii/S163107051630127X.

[18] Chin, C., Grimm, R., Julienne, P. & Tiesinga, E. Feshbach resonances in ultracold gases. *Reviews of Modern Physics* **82**, 1225–1286 (2010). URL https://link.aps.org/doi/10.1103/RevModPhys.82.1225.

[19] Zhao, E., Löfwander, T. & Sauls, J. A. Heat transport through Josephson point contacts. *Physical Review B* **69**, 134503 (2004). URL https://link.aps.org/doi/10.1103/PhysRevB.69.134503.

[20] Valtolina, G. *et al.* Josephson effect in fermionic superfluids across the BEC-BCS crossover. *Science* **350**, 1505–1508 (2015). URL https://www.science.org/doi/10.1126/science.aac9725.

[21] Luick, N. *et al.* An ideal Josephson junction in an ultracold two-dimensional Fermi gas. *Science* **369**, 89–91 (2020). URL https://www.science.org/doi/10.1126/science.aaz2342.

[22] Martín-Rodero, A., García-Vidal, F. J. & Levy Yeyati, A. Microscopic theory of Josephson mesoscopic constrictions. *Physical Review Letters* **72**, 554–557 (1994). URL https://link.aps.org/doi/10.1103/PhysRevLett.72.554.
[23] Cuevas, J. C., Martín-Rodero, A. & Yeyati, A. L. Hamiltonian approach to the transport properties of superconducting quantum point contacts. *Physical Review B* **54**, 7366–7379 (1996). URL https://link.aps.org/doi/10.1103/PhysRevB.54.7366.
[24] Meier, F. & Zwerger, W. Josephson tunneling between weakly interacting Bose-Einstein condensates. *Physical Review A* **64**, 033610 (2001). URL https://link.aps.org/doi/10.1103/PhysRevA.64.033610.
[25] Yao, J., Liu, B., Sun, M. & Zhai, H. Controlled transport between Fermi superfluids through a quantum point contact. *Physical Review A* **98**, 041601 (2018). URL https://link.aps.org/doi/10.1103/PhysRevA.98.041601.
[26] Öttinger, H. C. *Beyond equilibrium thermodynamics* (Wiley-Interscience, Hoboken, N.J., 2005).
[27] Häusler, S. *et al.* Interaction-Assisted Reversal of Thermopower with Ultracold Atoms. *Physical Review X* **11**, 021034 (2021). URL https://link.aps.org/doi/10.1103/PhysRevX.11.021034.
[28] Husmann, D. *et al.* Connecting strongly correlated superfluids by a quantum point contact. *Science* **350**, 1498–1501 (2015). URL https://science.sciencemag.org/content/350/6267/1498.
[29] Huang, M.-Z. *et al.* Superfluid Signatures in a Dissipative Quantum Point Contact. *Physical Review Letters* **130**, 200404 (2023). URL https://link.aps.org/doi/10.1103/PhysRevLett.130.200404.
[30] Agraït, N., Yeyati, A. L. & van Ruitenbeek, J. M. Quantum properties of atomic-sized conductors. *Physics Reports* **377**, 81–279 (2003). URL https://www.sciencedirect.com/science/article/pii/S0370157302006336.
[31] Viljas, J. K. Multiple Andreev reflections in weak links of superfluid $^3\mathrm{He}-B$. *Physical Review B* **71**, 064509 (2005). URL https://link.aps.org/doi/10.1103/PhysRevB.71.064509.
[32] Krinner, S., Stadler, D., Husmann, D., Brantut, J.-P. & Esslinger, T. Observation of quantized conductance in neutral matter. *Nature* **517**, 64 (2014). URL https://www.nature.com/articles/nature14049.
[33] Kwon, W. J. *et al.* Strongly correlated superfluid order parameters from dc Josephson supercurrents. *Science* **369**, 84–88 (2020). URL https://www.science.org/doi/10.1126/science.aaz2463.
[34] Ku, M. J. H., Sommer, A. T., Cheuk, L. W. & Zwierlein, M. W. Revealing the Superfluid Lambda Transition in the Universal Thermodynamics of a Unitary Fermi Gas. *Science* **335**, 563–567 (2012). URL http://science.sciencemag.org/content/335/6068/563.
[35] Haussmann, R. & Zwerger, W. Thermodynamics of a trapped unitary Fermi gas. *Physical Review A* **78**, 063602 (2008). URL https://link.aps.org/doi/10.1103/PhysRevA.78.063602.
[36] Haussmann, R., Rantner, W., Cerrito, S. & Zwerger, W. Thermodynamics of the BCS-BEC crossover. *Physical Review A* **75**, 023610 (2007). URL https://link.aps.org/doi/10.1103/PhysRevA.75.023610.
[37] Brantut, J.-P. *et al.* A Thermoelectric Heat Engine with Ultracold Atoms. *Science* **342**, 713–715 (2013). URL http://science.sciencemag.org/content/342/6159/713.
[38] Krinner, S. *et al.* Mapping out spin and particle conductances in a quantum point contact. *Proceedings of the National Academy of Sciences* **113**, 8144–8149 (2016). URL http://www.pnas.org/content/113/29/8144.
[39] Rammelmüller, L., Loheac, A. C., Drut, J. E. & Braun, J. Finite-Temperature Equation of State of Polarized Fermions at Unitarity. *Physical Review Letters* **121**, 173001 (2018). URL https://link.aps.org/doi/10.1103/PhysRevLett.121.173001.
[40] Sommer, A., Ku, M., Roati, G. & Zwierlein, M. W. Universal spin transport in a strongly interacting Fermi gas. *Nature* **472**, 201–204 (2011). URL https://www.nature.com/articles/nature09989.
[41] Fenech, K. *et al.* Thermodynamics of an Attractive 2D Fermi Gas. *Physical Review Letters* **116**, 045302 (2016). URL https://link.aps.org/doi/10.1103/PhysRevLett.116.045302.
[42] Boettcher, I. *et al.* Equation of State of Ultracold Fermions in the 2D BEC-BCS Crossover Region. *Physical Review Letters* **116**, 045303 (2016). URL https://link.aps.org/doi/10.1103/PhysRevLett.116.045303.
[43] Ketterle, W. & Zwierlein, M. W. Making, probing and understanding ultracold Fermi gases. *arXiv:0801.2500 [cond-mat]* (2008). URL http://arxiv.org/abs/0801.2500.
[44] Horikoshi, M. *et al.* Appropriate Probe Condition for Absorption Imaging of Ultracold ^{6}Li Atoms. *Journal of the Physical Society of Japan* **86**, 104301 (2017). URL http://journals.jps.jp/doi/

abs/10.7566/JPSJ.86.104301.
[45] Hueck, K. *et al.* Calibrating high intensity absorption imaging of ultracold atoms. *Optics Express* **25**, 8670–8679 (2017). URL https://opg.optica.org/oe/abstract.cfm?uri=oe-25-8-8670.
[46] Ockeloen, C. F., Tauschinsky, A. F., Spreeuw, R. J. C. & Whitlock, S. Detection of small atom numbers through image processing. *Physical Review A* **82**, 061606 (2010). URL https://link.aps.org/doi/10.1103/PhysRevA.82.061606.
[47] Reinaudi, G., Lahaye, T., Wang, Z. & Guéry-Odelin, D. Strong saturation absorption imaging of dense clouds of ultracold atoms. *Optics Letters* **32**, 3143–3145 (2007). URL https://www.osapublishing.org/abstract.cfm?uri=ol-32-21-3143.
[48] Ku, M. J.-H. *Thermodynamics and solitonic excitations of a strongly-interacting Fermi gas*. Thesis, Massachusetts Institute of Technology (2015). URL http://dspace.mit.edu/handle/1721.1/99309.
[49] Zürn, G. *et al.* Precise Characterization of ^{6}Li Feshbach Resonances Using Trap-Sideband-Resolved RF Spectroscopy of Weakly Bound Molecules. *Physical Review Letters* **110**, 135301 (2013). URL https://link.aps.org/doi/10.1103/PhysRevLett.110.135301.
[50] Veyron, R. *et al.* Quantitative absorption imaging: The role of incoherent multiple scattering in the saturating regime. *Physical Review Research* **4**, 033033 (2022). URL https://link.aps.org/doi/10.1103/PhysRevResearch.4.033033.
[51] Kazantsev, A. P., Surdutovich, G. I. & Yakovlev, V. P. *Mechanical action of light on atoms* (World Scientific Publ, Singapore, 1990).
